# Design of Optical Transducer for Recognition of Biomolecular Interactions between Bacterial Lipopolysaccharides and Amino Acids

**DOI:** 10.1002/advs.202523658

**Published:** 2026-03-12

**Authors:** Yena Choi, Sangmin Lee, Jin‐Kang Choi, Hyunsoo Han, Yeongseon Choi, Jun‐Hyung Im, Hyein Kim, Sangmin Jeon, Minjae Lee, Chang Yun Son, Young‐Ki Kim

**Affiliations:** ^1^ Department of Chemical Engineering Pohang University of Science and Technology (POSTECH) Pohang Republic of Korea; ^2^ Department of Chemistry Seoul National University Seoul Republic of Korea; ^3^ Department of Energy Science & Engineering, Department of Chemistry Kunsan National University Gunsan Republic of Korea

**Keywords:** amino acids, aspartic acids, biomolecular interactions, glutamic acids, liquid crystals, *salmonella*

## Abstract

Recognition of biomolecular phenomena at interfaces is a key scientific challenge with fundamental and practical importance. In this work, we discover two structurally similar amino acids, glutamic acid (Glu) and aspartic acid (Asp), showing different biological functions, have a significant but distinct orientational coupling with liquid crystals (LCs). Combining experiments with density functional theory calculations, we reveal that Glu and Asp exhibit unique adsorption‐desorption dynamics at the LC‐aqueous interfaces, producing distinct macroscopic optical signals. These interfacial behaviors are specifically governed by pH condition of the aqueous solution because the pH variations cause significant changes in charge states of the amino acids and thus their intermolecular interactions with LCs. Additionally, the dynamics are also modulated by the presence of other biological molecules (e.g., bacterial byproducts of *Salmonella* lipopolysaccharide, *Staphylococcus aureus* lipoteichoic acid, and *Escherichia coli* lipopolysaccharide) forming complexes with the amino acids, which dramatically change the LC's optical response. Leveraging this specific recognition mechanism for the interfacial dynamics of biomolecules and their interactions with other biological species, we also demonstrate the practical LC systems that autonomously recognize and optically report *Salmonella* at trace concentrations (10^2^ cfu/ml) within 1 min, significantly faster than the conventional methods (PCR and ELISA) requiring several hours.

## Graphical abstract

A deep comprehension of biomolecular phenomena at interfaces is significant with their fundamental and practical importance. Our experimental and theoretical investigations reveal that specific amino acids (glutamic acid and aspartic acid) exhibit an orientational coupling with liquid crystals, which can recognize and optically report the interfacial dynamics of biomolecules and their interactions with other biological species, such as bacterial byproducts.

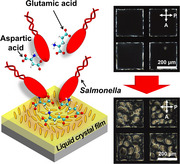

## 1 Introduction

Biomolecular phenomena occurring at interfaces are ubiquitous in biological systems, as found in membrane dynamics [1, 2] (e.g., selective transport of biomolecules, cell division), immune responses [3] (e.g., antigen‐antibody reactions between immune cells and pathogen), and cell signaling [4] (e.g., signal transduction for metabolic reprogramming upon binding of insulin with receptors at the surface of T cells). Recognition of such interfacial events is critical not only for fundamental understanding of biomolecular interactions [5, 6, 7] but also for offering the basis for designing practical applications in a variety of fields such as cell‐based assays, cell signaling analysis, screening of biomarkers, and biosensors [8, 9]. Accordingly, past studies have attempted to elucidate the underlying working principles for interfacial behavior of biomolecules through a range of techniques such as surface plasmon resonance, infrared spectroscopy, and reflectometric interference spectroscopy [8, 9]. Nevertheless, deciphering the biomolecular interplays at interfaces still remains a central scientific challenge. In this context, liquid crystal (LC), an intermediate phase of matter between an isotropic liquid and a crystalline solid, has offered promising platforms to design simple and versatile recognition systems for biomolecular events. The unique combination of fluidity, long‐range molecular ordering, and anisotropic characteristics (e.g., birefringence, dielectricity, elasticity) [10, 11, 12, 13] enables the LCs to sensitively respond to changes in their environment, including molecular‐ and nanoscopic‐scale events (e.g., interfacial adsorption of biomolecules), and then induce a local perturbation of LC orientation [14, 15, 16, 17, 18]. Subsequently, LCs amplify the localized LC reorientation into a macroscopic LC ordering transition that is transduced to optical outputs even visible to the naked eye. The outstanding stimuli‐responsiveness and amplification ability of LCs have been widely utilized to optically recognize the intermolecular interactions and interfacial behaviors of biomolecules. For example, a variety of biomolecules (e.g., DNA, RNA, protein, glucose, lipid, enzyme, endotoxin, bacterial lipopolysaccharide) were demonstrated to interact with the LC interfaces and trigger the ordering transition (and thus the optical transition) of LCs [15, 19, 20, 21, 22, 23, 24]. Beyond equilibrium, the LCs are also capable of optically reporting dynamic behaviors of biological species, such as the motion of bacteria [16], the collision of supramolecular assemblies [14], protein folding [25], and enzymatic activity [22]. Furthermore, the LCs can be programmed to recognize a target biological event with an excellent level of selectivity (e.g., specific binding of proteins and viruses) and sensitivity (e.g., optical transition of LCs upon interfacial adsorption of trace concentrations of endotoxin as low as < 1pg/ml), respectively, through interfacial treatments of LCs with aptamer and synthetic amphiphiles [19, 20, 26], and the use of droplet geometry where a large elastic strain is stored [15].

In this work, we discover two specific biomolecules, the amino acids glutamic acid (Glu) and aspartic acid (Asp) which are structurally very similar yet possess different biological functions in biological tissues [27, 28], to have a significant but distinct orientational coupling with LCs, allowing us to design the LC‐based optical transducer to recognize dynamic interfacial behaviors of Glu/Asp and their biomolecular interactions with other biological species. Specifically, we find Glu and Asp in an aqueous solution to undergo spontaneous adsorption and subsequent desorption processes at the LC interfaces and cause a transient orientational transition of interfacial LC molecules, which is transduced into a macroscopic optical signal alternating bright (for adsorption) and dark states (for desorption). Combining experiments with density functional theory (DFT) calculations, the interfacial adsorption‐desorption dynamics of Glu/Asp and their interplay with LCs are demonstrated to be governed by pH conditions because the pH variations cause changes in the charge states of the amino acids and thus their intermolecular interactions with LCs. Additionally, we reveal that Glu and Asp electrostatically interact with other biological molecules, such as bacterial byproducts of lipopolysaccharides (LPS) and lipoteichoic acid (LTA) to form biomolecular complexes, as validated by the energy decomposition analysis. These complexes significantly change the interfacial behavior of Glu/Asp and thus the orientational coupling with LCs by promoting their adsorption and stabilization at the LC interface, the dynamics of which depend on the characteristics of biomolecules binding to the amino acids (e.g., charged functional moieties, the number of aliphatic tails). Therefore, the LC systems can recognize specific interfacial behaviors of amino acids, which is manipulated by pH conditions and the presence of other biological species, and report them through distinct macroscopic optical signals, such as transient/persistent optical transitions, different transition rates, and varying optical intensities. Finally, building on our deep fundamental understanding of the correlative interplay of LCs with the amino acids and their complexes with other biological species, we demonstrate the practical potential of LC systems to autonomously recognize and optically report the presence of *Salmonella* cells at trace concentration (10^2^ cfu/ml, 10% level of infective dose) with a recognition time of less than 1 min, which is significantly faster than the conventional methods (polymerase chain reaction (PCR) and enzyme‐linked immunosorbent assay (ELISA)) requiring an assay time of at least 6 h [29].

## 2 Results and Discussion

### 2.1 LC‐Based Optical Transducer for Specific Biomolecular Events

**FIGURE 1:**
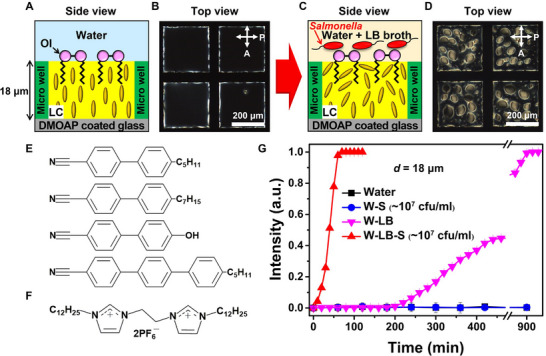
Liquid crystal‐based optical transducer to autonomously recognize and optically report the presence of specific biomolecules in overlying aqueous solutions. (A‐D) (A, C) Schematic illustrations of the liquid crystal (LC) system with a reconstructed orientation of LC molecules and (B, D) corresponding polarizing optical micrographs (A, B) before and (C, D) after the introduction of Luria‐Bertani (LB) broth containing *Salmonella* into the overlying water phase. The thickness *d* of the LC film is 18 µm. The LC‐aqueous interface is decorated with **OI‐C12** in (F). The yellow and red ellipsoidal rods represent E7 molecules and *Salmonella*, respectively. White letters P and A in (B) and (D) indicate the orientations of the polarizer and analyzer, respectively. The micrographs in (B) and (D) are taken at 50 min after the incubation. (E) Molecular structures of the nematic LC mixture, E7. (F) Molecular structure of the organic ionic plastic crystal with two hydrophobic tails of a twelve‐carbon alkyl chain (**OI‐C12**). (G) Intensity profiles of the LC films (*d* = 18 µm, with **OI‐C12**) upon incubation in pure water (black line) and aqueous solutions with *Salmonella* (W‐S solution, blue line), LB broth (W‐LB solution, pink line), or both (W‐LB‐S solution, red line). The equilibrium concentration of LB broth (*C*_LB_) and *Salmonella* (*C_S_*) in the overlying aqueous solution are *C*_LB_ = 16.7% (v/v) and *C_S_* ∼ 10^7^ cfu/ml, respectively.

Figure 1A illustrates the representative geometry of micro‐thick LC film with a vertical (homeotropic) molecular orientation that can sensitively recognize a range of biological phenomena occurring at the LC‐aqueous interface (e.g., motion of bacteria, adsorption of biomolecules) and transduce them into macroscopic optical outputs [16, 20, 30, 31]. The overlying aqueous phase allows for facile delivery of biological species to the LC interface [31, 32]. The micro‐thick LC films (thickness *d* = 18 µm) are prepared by loading a nematic LC (E7, Figure 1E) into micro‐wells hosted on a glass substrate and then submerging them into aqueous solutions. The homeotropic LC ordering (Figure 1A) is achieved by chemically treating the glass substrate and the LC‐aqueous interface, respectively, with dimethyloctadecyl 3‐(trimethoxysilyl)propyl ammonium chloride (DMOAP) [14, 20] and an organic ionic plastic crystal (**OI**) bearing two hydrophobic tails of twelve‐carbon alkyl chain (**OI‐C12**, Figure 1F) [33], as demonstrated by a dark optical texture between crossed‐polarizers under polarizing optical microscopy (POM, Figure 1B). Specifically, the glass substrate is coated with DMOAP via dipping method [14, 20] and the LC‐aqueous interface is decorated through the self‐assembly of **OI‐C12** that is initially doped into the LC with a concentration of 0.2 mM. It is worth mentioning thatto impose the homeotropic anchoring of LCs at the aqueous interface, we deliberately select **OI‐C12**, a new class of functional molecules for LCs [33], rather than widely used conventional surfactants such as sodium dodecyl sulfate (SDS) and dodecyltrimethylammonium bromide (DTAB) [31, 34, 35] because of the following two reasons. First, unlike SDS and DTAB [36, 37], the extremely low water solubility of **OI‐C12** prevents their desorption from the LC interface to the overlying water phase. Therefore, when contacted with a water phase, the **OI‐C12**‐laden LC interface can maintain the homeotropic anchoring for a long period of time, while the SDS‐ or DTAB‐decorated LC interface cannot (Figure S1). Second, due to no desorption of **OI‐C12** from the LC‐aqueous interface, one can circumvent any unwanted chemical or biological reactions between the desorbed functional molecules and analytes present in the overlying water phase (e.g., complex formation of surfactants, cell death) [38, 39, 40, 41], which can preclude an unambiguous interpretation.

In this condition, we observe that the LC film can maintain the homeotropic configuration under water incubation over a time period of a few days, reflected by no sign of changes in the optical intensity of LC film from the dark state (Figure 1B and black line in Figure 1G). Surprisingly, however, when a cell culture medium of Luria‐Bertani (LB) broth containing *Salmonella* is introduced into the overlying water phase (Figure 1C), birefringent domains appear at the LC‐aqueous interface within 10 min and gradually grow for 50 min (Figure 1D and red line in Figure 1G). The equilibrium concentrations of LB broth (*C*_LB_) and *Salmonella* (*C_S_*) in the overlying aqueous solution are *C*_LB_ = 16.7% (v/v) and *C_S_* ∼ 10^7^ cfu/ml, respectively. To uncover the underlying mechanism for the emergence of bright domains indicative of reorientation of LC molecules at the aqueous interface, we investigate the orientational coupling of potential key determinants (LB broth and *Salmonella*) with the LC. To this end, as shown in Figure 1G, we measure the temporal evolution of optical intensity from the LC films between crossed‐polarizers under the incubation in water with LB broth (*C*_LB_ = 16.7% (v/v), W‐LB solution) or *Salmonella* (*C_S_* ∼ 10^7^ cfu/ml, W‐S solution), and then compare them with the result from the aqueous solution containing both LB broth and *Salmonella* (W‐LB‐S solution). In the W‐S solution (*C_S_* ∼ 10^7^ cfu/ml), the LC films show no optical transitions over 900 min (blue line in Figure 1G), consistent with the observation under water incubation (black line in Figure 1G). When the LC films are incubated into the W‐LB solution (*C*_LB_ = 16.7% (v/v)), no optical transition from the initial dark state is also observed for more than 3 h (pink line in Figure 1G). After 200 min of incubation, however, the homeotropic LC orientation starts to be perturbed at the aqueous interface, reflected by the appearance of bright domains leading to an increase in the optical intensity. These results imply that some components in the LB broth are gradually adsorbed at the LC‐aqueous interface and then drive the orientational transition of LCs (Figure 1C) from the initial homeotropic configuration (Figure 1A), which is accompanied by the dark‐to‐bright optical transition (Figure 1D). In particular, the transition is significantly accelerated in the W‐LB‐S solution (*C*_LB_ = 16.7% (v/v) and *C_S_* ∼ 10^7^ cfu/ml), under which the LC films exhibit the macroscopic optical signal within 10 min (red line in Figure 1G). It is important to point out this fast recognition of the presence of *Salmonella* cells by the LC film. Given that the conventional methods such as PCR and ELISA require more than 6 h for detecting the presence of *Salmonella* cells at *C_S_* ∼ 10 cfu/ml (1% level of infective dose for typhoid fever) [29, 42], our LC system shows a considerably faster detection time less than 5 h even before any optimizations. This efficiency is possible because the utilized *C_S_* ∼ 10^7^ cfu/ml is achievable by culturing 10 cfu/ml of *Salmonella* cells for 4.5 h at typical culturing temperature (*T* = 37 °C) or even less time at higher *T*. Furthermore, we demonstrate that our LC system can be designed to achieve a significantly reduced recognition time (< 0.5 h) by enhancing its sensitivity (*C_S_* ∼ 10^2^ cfu/ml), which will be discussed in detail in Figure 6.

### 2.2 Adsorption‐Desorption Dynamics of Glu and Asp at the LC‐Aqueous Interface

**FIGURE 2:**
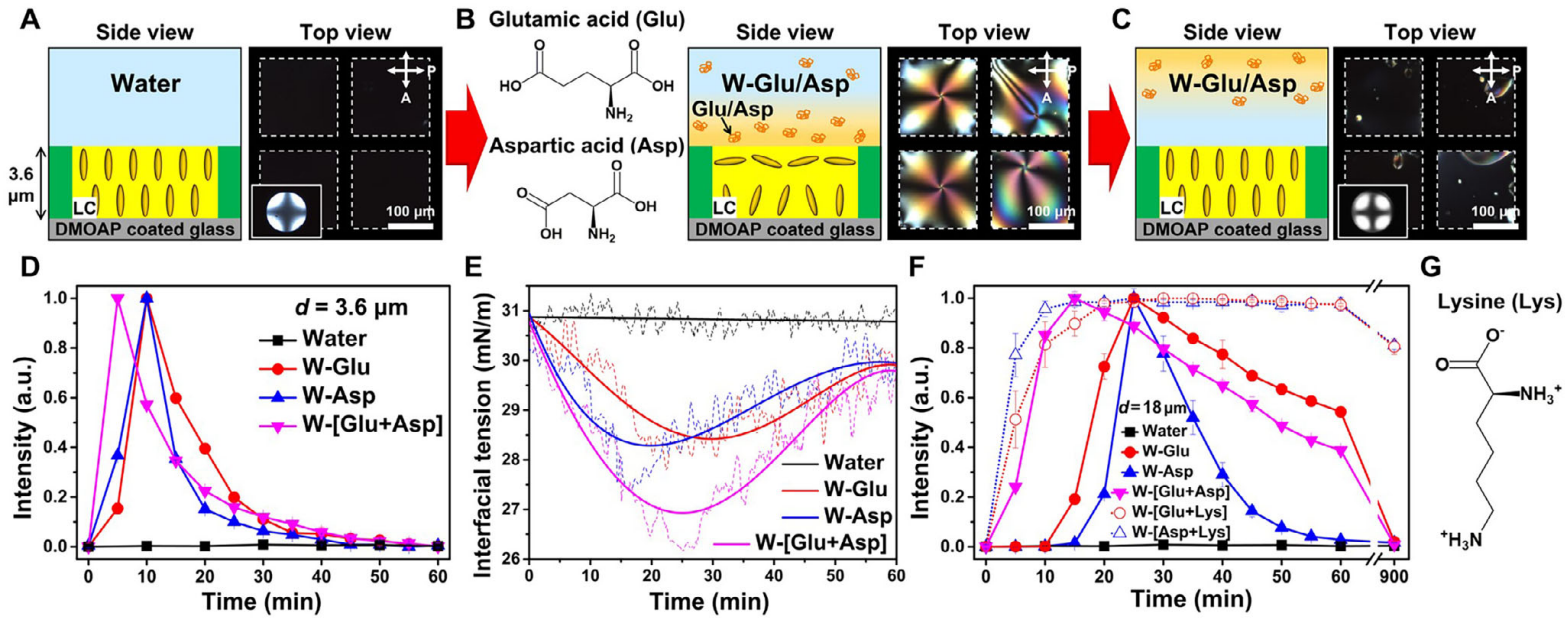
Molecular reorientations and accompanying optical transitions of the LC films in response to interfacial adsorption and desorption of glutamic acid (Glu), aspartic acid (Asp), and lysine (Lys). (A–C) Schematic illustrations of the thin LC films (*d* = 3.6 µm, no **OI‐C12**) with reconstructed LC ordering and corresponding POM images (A) before and (B, C) after the introduction of Glu/Asp at (B) 10 min and (C) 1 h. (D) Corresponding intensity profiles of the thin LC films upon incubation in pure water (black line) and aqueous solutions with Glu (W‐Glu solution, red line), Asp (W‐Asp solution, blue line), or both (W‐[Glu+Asp] solution, pink line). The concentrations of Glu (*C*_Glu_ = 3.2 mM) and Asp (*C*_Asp_ = 1.2 mM) are equivalent to those in the W‐LB solution of *C*_LB_ = 16.7% (v/v). (E) Dynamic interfacial tension (*σ*) measured at the LC‐aqueous interfaces in the presence of Glu and Asp in the aqueous solutions. (F) Intensity profiles of the thick LC films (*d* = 18 µm, with **OI‐C12**) upon the incubation in pure water (solid‐black line) and the solutions of W‐Glu (solid‐red line), W‐Asp (solid‐blue line), W‐[Glu+Asp] (solid‐pink line), W‐[Glu+Lys] (dotted‐red line), and W‐[Asp+Lys] (dotted‐blue line). The concentration of Lys (*C*_Lys_ = 1.1 mM) is equivalent to that in the W‐LB solution of *C*_LB_ = 16.7% (v/v). (G) Molecular structure of lysine.

In order to identify components of the LB broth (sodium chloride, tryptone, and yeast extract) [43] that trigger the anchoring transition of LCs at the aqueous interface, we characterize intensity changes of the homeotropic LC films upon introduction of each component into the overlying water phase (Figure S2). The injected concentration of each component is equivalent to that in the W‐LB solution (*C*_LB_ = 16.7% (v/v), pink line in Figure 1G). In this experiment (Figure 2A–D), we use the LC‐aqueous interface with no treatment of **OI‐C12** to omit potential complex interactions of the introduced components with **OI‐C12** and thus to define the interfacial coupling of LCs only to the components. Because E7 assumes a tangential anchoring at the interface of pure water without **OI‐C12**, however, the LC film adopts a hybrid configuration like Figure 1C (i.e., the tangential anchoring at the LC‐aqueous interface and the homeotropic anchoring at the DMOAP‐coated glass substrate). Therefore, we induce the homeotropic configuration by reducing *d* from 18 to 3.6 µm (Figure 2A) under which the thin LC film (*d* = 3.6 µm, no **OI‐C12**) promotes the tangential‐to‐homeotropic anchoring transition at the aqueous interface to minimize the elastic bulk free energy because the hybrid configuration causes a large elastic strain within the thin LC film [44]. We measure the LC‐aqueous interface of thin LC films to maintain the homeotropic anchoring over several hours, reflected by no optical changes from the dark state under POM (Figure 2A), the Maltese‐cross pattern under conoscopy (inset in Figure 2A), and the absence of optical intensity (black line in Figure 2D).

**FIGURE 3:**
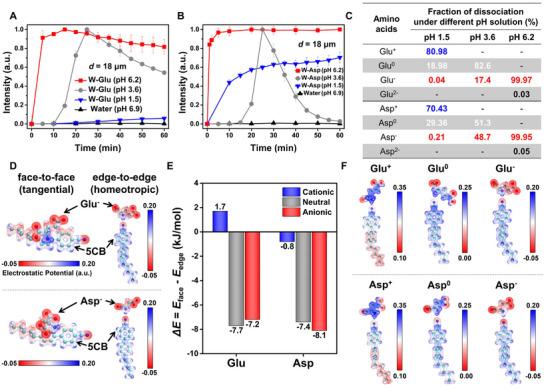
pH‐dependent optical response of the LC films and orientational coupling of LCs with Glu and Asp simulated by density functional theory (DFT) calculations. (A, B) Intensity profiles of the thick LC films (*d* = 18 µm, with **OI‐C12**) upon incubation in (A) the W‐Glu and (B) W‐Asp solutions at different pH conditions (pH 1.5, 3.6, and 6.2). (C) Fractions of dissociation species of Glu and Asp in aqueous solutions at pH 1.5, pH 3.6, and pH 6.2. (D) Optimized geometries (face‐to‐face and edge‐to‐edge) and corresponding electrostatic potential (ESP) maps for the binding of Glu^–^ and Asp^–^ to the nematic LC molecule of 4‐cyano‐4’‐pentylbiphenyl (5CB, a major component of E7) simulated by DFT calculations. ESP map isosurfaces are rendered at an electron density of 0.04 a.u. (E) DFT‐calculated relative stabilization energies (kJ/mol), Δ*E* = *E*_face_—*E*_edge_ for the binding of 5CB with cationic, neutral, and anionic forms of Glu/Asp in aqueous solutions. (F) DFT‐calculated ESP maps for the binding of 5CB with Glu^+^/Asp^+^, Glu^0^/Asp^0^, and Glu^–^/Asp^–^.

Among various components in the LB broth, we find that two specific amino acids (Figure 2B), glutamic acid (Glu) and aspartic acid (Asp), play a critical role in the homeotropic‐to‐tangential anchoring transition at the LC‐aqueous interface, a phenomenon that has yet to be reported. In the aqueous solutions containing either Glu (W‐Glu solution, *C*_Glu_ = 3.2 mM) or Asp (W‐Asp solution, *C*_Asp_ = 1.2 mM), we observe that the homeotropic thin LC film (Figure 2A) exhibits a gradual dark‐to‐bright optical transition over the time perod o 10 min (Figure 2B and the red and blue lines in Figure 2D). This transition implies that Glu and Asp adsorb at the LC‐aqueous interface and drive the anchoring transition from the initial homeotropic orientation (the pH‐dependent charge states of Glu/Asp and their interfacial behavior will be discussed in detail in Figure 3). In contrast to our observation from the thick LC film (*d* = 18 µm, with **OI‐C12**) in the W‐LB solution (pink line in Figure 1G), however, the perturbed LC orientation gradually restores the initial homeotropic anchoring from 10 min of incubation (Figure 2C), suggesting the desorption of Glu and Asp from the LC‐aqueous interface. Moreover, the transition and restoration processes are accelerated in the presence of both Glu and Asp in the overlying water phase (W‐[Glu+Asp] solution, pink line in Figure 2D). To confirm whether the adsorption and desorption of Glu/Asp occur at the LC‐aqueous interface, therefore, we measure dynamic interfacial tension (*σ*) of the LC‐aqueous interface. Consistent with previous studies [41], *σ* of the E7‐water interface is measured to be 30.8 ± 0.1 mN/m and remains stable over few hours (black line in Figure 2E). Upon the introduction of Glu, Asp, or both into the water phase, however, we observe *σ* to notably decrease for the initial 20∼30 min and then increase again to 29.9 ± 0.6 mN/m (red, blue, and pink lines in Figure 2E). The measured dynamics of *σ* corroborate that the transient homeotropic‐to‐tangential anchoring transition (Figure 2A–C) results from the adsorption and subsequent desorption processes of Glu and Asp at the LC‐aqueous interface.

Based on our findings regarding the interplay between the interfacial orientation of LCs and the adsorbates (Glu and Asp), we now return to the original LC system (i.e., thick LC film of *d* = 18 µm with **OI‐C12** treatment, Figure 1A). Interestingly, in the W‐Glu, W‐Asp, and W‐[Glu+Asp] solutions, the thick LC film also exhibits the transient optical transition (solid‐red, ‐blue, and ‐pink lines in Figure 2F), analogous to the results in the thin LC film (Figure 2D). Therefore, the persistent bright state observed in the W‐LB solution (Figure 1D and pink line in Figure 1G) suggests that there exist components in the LB broth to preclude the desorption of Glu and Asp from the LC‐aqueous interface. We determine the cationic amino acid, lysine (Lys, Figure 2G), to be the key component for stabilizing the adsorption of Glu and Asp at the LC‐aqueous interface. When Lys (*C*_Lys_ = 1.1 mM) is present in the W‐Glu and W‐Asp solutions (dotted‐red and ‐blue lines in Figure 2F), we measure that the intensity of the thick LC films does not return to the initial dark state but is stabilized in a bright state with birefringent domains. We attribute these interfacial phenomena to (i) the strong electrostatic affinity of Lys with anionic Glu and Asp, leading to the formation of Lys‐Glu and Lys‐Asp complexes [45, 46] and (ii) the presence of hydrophobic aliphatic tail in Lys, which favors to interdigitate into LCs [14, 15, 16]. In particular, it was demonstrated that the presence of linear aliphatic tails in complexes promotes their adsorption at the LC‐aqueous interface and thus contributes to hindering their desorption [31, 35], consistent with our observation regarding the persistent bright state (dotted lines in Figure 2F).

It is also worth noting that although Glu and Asp are structurally very similar (differing by only one extra methylene group in Glu, Figure 2B), the thick LC film (*d* = 18 µm, with **OI‐C12**) exhibits distinct intensity profiles in the W‐Glu (solid‐red line in Figure 2F) and W‐Asp (solid‐blue line in Figure 2F) solutions, which allows for their clear optical differentiation. The identification between Glu and Asp is highly important in biochemical and medical fields due to their distinct functions in biological tissues. For instance, they contribute differently to neurotransmission (e.g., Glu serves as the precursor of γ‐aminobutyric acid, while Asp is the precursor for *N*‐acetylaspartate), and Asp acts as an independent biomarker for diabetic retinopathy [27, 28]. However, distinguishing between them remains a challenging task for conventional methods, which often require complicated equipment and lengthy processes [47]. Therefore, the results in Figure 2F also demonstrate that our LC system provides a simple and versatile tool to identify the type of adsorbates, thereby facilitating a deeper understanding of biological metabolism and advancing theragnostic applications.

### 2.3 pH‐Dependent Interfacial Dynamics of Glu/Asp and Their Orientational Coupling with LCs

In addition to the contribution of Lys, we also find that the pH condition of the overlying aqueous solution plays an important role in the adsorption and desorption processes of Glu and Asp at the LC‐aqueous interface. While the W‐S, W‐LB, and W‐LB‐S solutions (Figure 1) show a pH of 6.8 that slightly differs from the pH value of pure water (pH 6.9), we measure the W‐Glu, W‐Asp, and W‐[Glu+Asp] solutions (Figure 2) to show considerably low pH 3.6 ± 0.05. Because such significant pH variation was shown to drive a change in the dissociation fraction of amino acids [48, 49], we investigate how the pH condition influences the interfacial adsorption‐desorption dynamics of Glu/Asp and thus the anchoring transition of LCs. To this end, we add Glu (*C*_Glu_ = 3.2 mM) or Asp (*C*_Asp_ = 1.2 mM) into different aqueous pH buffers with the initial pH values of 4, 7, and 10, leading to the equilibrium pH values of 1.5, 3.6, and 6.2, respectively. In the W‐Glu (Figure 3A) and W‐Asp (Figure 3B) buffer solutions, the homeotropic thick LC films (*d* = 18 µm, with **OI‐C12**) exhibit different anchoring transition behaviors depending on the solutions’ pH values. We note that any optical transitions are not observed from the thick LC films in the aqueous pH buffer solutions without Glu and Asp regardless of pH values (Figure S3). We find the intensity profiles measured from the thick LC films at low pH 1.5 (blue lines in Figure 3A,B) and high pH 6.2 (red lines in Figure 3A,B) to be distinct from those at pH 3.6 showing the transient optical transition (gray lines in Figure 3A,B). Specifically, at low pH 1.5 and high pH 6.2 conditions, the induced bright states appear to be stabilized, and the initial homeotropic anchoring is not restored, indicating the interfacial desorption of Glu and Asp to be hindered. Moreover, the maximum intensities at pH 1.5 are measured to be lower than those at pH 3.6 and 6.2 (especially in the W‐Glu solutions), suggesting that the interfacial adsorption of Glu and Asp is hampered at the low pH condition.

To uncover these pH dependencies, we calculate the fraction of dissociations in the W‐Glu and W‐Asp solutions at pH 1.5, 3.6, and 6.2 (see Methods) [50]. As shown in Figure 3C, at low pH 1.5, positively charged Glu^+^ (80.98%, Figure S4A) and Asp^+^ (70.43%, Figure S4B) are dominantly present followed by neutrally charged Glu^0^ (18.98%, Figure S4C) and Asp^0^ (29.36%, Figure S4D), and only small quantities of Glu^–^ (0.04%, Figure S4E) and Asp^–^ (0.21%, Figure S4F). As the pH increases to pH 3.6 (which is equivalent to the pH condition of the W‐Glu, W‐Asp, and W‐[Glu+Asp] solutions wherein the transient optical transition of LCs is observed (Figure 2)), however, Glu^+^ and Asp^+^ are no longer produced from the dissociation of amino acids but the fraction of Glu^0^ (from 18.98% to 82.6%) and Asp^0^ (from 29.36% to 51.3%) is remarkably increased. Moreover, at the high pH 6.2 condition (which is close to the pH condition of the W‐S, W‐LB, and W‐LB‐S solutions (pH 6.8) wherein the persistent optical transition of LCs is observed (Figure 1)), Glu and Asp are fully negatively ionized to Glu^–^, Glu^2–^, Asp^–^, and Asp^2–^. Consequently, from combination of the intensity profiles (Figure 3A,B) and the calculated fraction of dissociations at different pH conditions (Figure 3C), we verify that (i) negatively charged Glu^–^ and Asp^–^ tend to be more preferentially adsorbed and stabilized at the LC‐aqueous interface than other species (red lines in Figure 3A,B), (ii) the neutrally charged Glu^0^ and Asp^0^ are responsible for the transient anchoring transition (i.e., desorption of Glu and Asp, gray lines in Figure 3A,B), and (iii) the interfacial adsorption of positively charged Glu^+^ (blue line in Figure 3A) is significantly weaker than that of Asp^+^ (blue line in Figure 3B).

To provide further insights into how the charge states of Glu and Asp govern their interactions with LCs at the molecular level, we carry out density functional theory (DFT) calculations for the anionic (Glu^–^/Asp^–^), neutral (Glu^0^/Asp^0^), and cationic (Glu^+^/Asp^+^) amino acids interacting with the nematic LC 4‐cyano‐4'‐pentylbiphenyl (5CB, a major component of E7, first row in Figure 1E). These calculations identify two stable binding modes, face‐to‐face and edge‐to‐edge (Figure 3D), which are associated with the tangential and homeotropic anchoring of LCs, respectively. In the face‐to‐face configuration, the optimized geometries and corresponding electrostatic potential (ESP) maps reveal that the benzene ring of 5CB produces a region of negative electrostatic potential on its surface, which strongly promotes a cation‐π interaction with the protonated amino group (‐NH_3_^+^) in Glu and Asp (Figure S4). In the edge‐to‐edge configuration, however, the ‐NH_3_^+^ group instead interacts with the nitrile (‐CN) moiety of 5CB through a dipole‐dipole interaction. To compare the stability of these two binding configurations, we define the relative stabilization energy as Δ*E* = *E*_face_—*E*_edge,_ where *E*_face_ and *E*_edge_ are the face‐to‐face and the edge‐to‐edge binding energies, respectively, and a negative Δ*E* value denotes a preference for the face‐to‐face (i.e., tangential) arrangement. In the aqueous phase, the calculated Δ*E* values are 1.7 kJ/mol for Glu^+^, −7.7 kJ/mol for Glu^0^, −7.2 kJ/mol for Glu^–^, −0.8 kJ/mol for Asp^+^, −7.4 kJ/mol for Asp^0^, and −8.1 kJ/mol for Asp^–^ (Figure 3E). Specifically, although *E*_face_ remains similar across all charge states, the edge‐to‐edge binding becomes progressively less favorable as the net charge of amino acids becomes more negative (Figure S5). This destabilization arises because the partially negative nitrile moiety of 5CB experiences increasing electrostatic repulsion from the negatively charged carboxylate group (‐COO^–^) in neutral Glu^0^/Asp^0^ and anionic Glu^–^/Asp^–^ (Figure 3F), a phenomenon that is particularly pronounced for Asp due to its shorter side‐chain. Collectively, the DFT results indicate that the anionic Glu^–^/Asp^–^, neutral Glu^0^/Asp^0^, and cationic Asp^+^ (∆*E* < 0, Figure 3E) preferentially adopt the face‐to‐face configuration with LCs and thereby are expected to trigger the ordering transition of LCs toward the tangential anchoring at the LC‐aqueous interface, but the cationic Glu^+^ (∆*E* > 0) energetically favors the edge‐to‐edge state (i.e., weak or no transition from homeotropic anchoring), which are in good qualitative agreement with our experiments.

**FIGURE 4:**
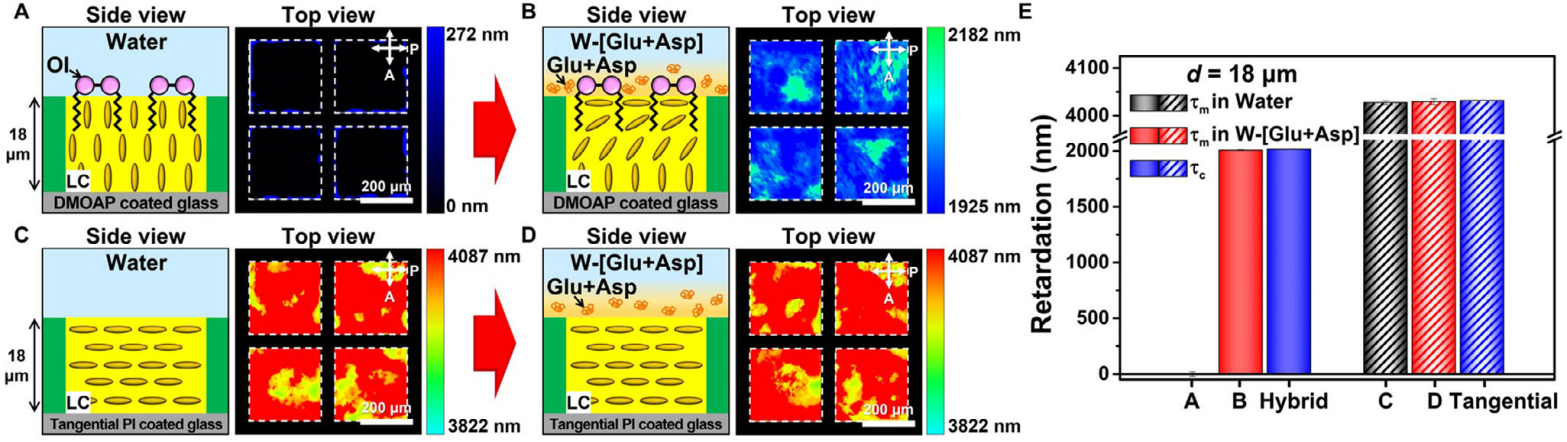
Amino acids‐induced easy axis reorientation of LCs revealed by retardation measurements from the LC films. (A‐D) Schematic illustrations (side view) of (A, B) the homeotropic (with **OI‐C12**) and (C, D) tangential thick LC films (no **OI‐C12**), and corresponding retardation color maps (top view) in (A, C) the pure water and (B, D) W‐[Glu+Asp] solutions. The retardation color maps in (B) and (D) are taken when the retardation reaches the maximum value. (E) Retardation values measured (*Γ*_m_, black and red bars) from (A‐D) and calculated (*Γ*_c_, blue bars) from LC films with the hybrid and tangential configurations corresponding to the reconstructed LC ordering in (B) and (D), respectively.

Because our findings regarding the significant orientational coupling of LCs with the amino acids of Glu/Asp and their complexes have not been investigated before, we perform additional experiments to reveal the underlying mechanism for the anchoring transition occurring at the LC‐aqueous interface induced by the adsorption of Glu and Asp. In our configuration, the transition from the initial homeotropic anchoring (i.e., the dark‐to‐bright optical transition) can occur through either (i) formation of isotropic LC domains at the LC‐aqueous interface that generates nematic‐isotropic interfaces at which E7 assumes a tilted molecular orientation [51, 52] or (ii) changes in easy axis of LCs at the aqueous interface [53, 54, 55]. To identify the underlying mechanism, we measure changes in optical retardation of the homeotropic thick LC films (*d* = 18 µm, with **OI‐C12**) in pure water (Figure 4A) and W‐[Glu+Asp] solutions (Figure 4B) [56]. In pure water, the measured average value of optical retardation (*Γ*_m_) remains unchanged from ∼ 0 nm (Figure 4A and solid‐black bar in Figure 4E), matching no transition from the initial homeotropic configuration (black line in Figure 1G). In the W‐[Glu+Asp] solution, however, the LC films exhibit a significant increase in *Γ*_m_ from 0 to 2009 ± 2 nm within 15 min (Figure 4B and solid‐red bar in Figure 4E), which then gradually decreases back to 0 nm (solid‐red bars in Figure S6). This behavior is consistent with their transient optical transition (solid‐pink line in Figure 2F), further supporting the interfacial adsorption and subsequent desorption of Glu and Asp. Importantly, we find that the maximum *Γ*_m_ = 2009 ± 2 nm at around 15 min in the W‐[Glu+Asp] solution closely matches the calculated retardation value of *Γ*_c_ = 2016 nm (solid‐blue bar in Figure 4E) from the thick LC film with the hybrid LC configuration (schematic in Figure 4B) [13, 57, 58]. This comparable match of retardation values indicates no formation of isotropic domains at the LC‐aqueous interfaces (mechanism i) because the presence of such isotropic domains would result in a maximum *Γ*_m_ lower than *Γ*_c_. Furthermore, upon temperature variations between 25°C and 55°C, we observe the measured *Γ*_m_ to closely match the *Γ*_c_ values calculated for the hybrid configuration using the temperature‐dependent birefringence (Δ*n*) of E7 (Figure S7A). This result indicates that our LC system remains stable, maintaining its hybrid configuration (i.e., a bright optical signal) even at elevated temperatures up to 55°C, which is near the nematic‐isotropic coexistence phase transition. It is important to note that while *Γ*_m_ and *Γ*_c_ are comparable across all investigated *T*, the deviation between the two values increases at higher *T* (Figure S7A). Given that *Γ*_m_ < *Γ*_c_, this observation suggests that a small amount of Glu and Asp likely desorb from the LC interface at higher temperatures. This desorption causes a subtle deviation of LCs from the tangential anchoring (i.e., a slightly tilted LC orientation), resulting in the observed minor reduction in *Γ*_m_.

For further confirmation of the anchoring transition mechanism, we also measure *Γ*_m_ from LC films having the tangential anchoring at both the aqueous and glass interfaces (Figure 4C). To achieve the tangential anchoring, the LC‐aqueous interface is not treated with **OI‐C12**, and the glass substrate is coated with a tangential polyimide layer and then rubbed. We observe that the *Γ*_m_ values of the tangential thick LC films in pure water (*Γ*_m_ = 4028 ± 2 nm, dashed‐black bar in Figure 4E) and in the W‐[Glu+Asp] solutions (*Γ*_m_ = 4030 ± 3 nm, dashed‐red bar in Figure 4E) are comparable. These values are also closely consistent with *Γ*_c_ = 4032 nm for an LC film with the tangential configuration (dashed‐blue bar in Figure 4E). The *Γ*_m_ value remains unaltered over the time period of 900 min (dashed‐red bars in Figure S6). This stability is attributed to the water‐induced tangential anchoring of LCs at the LC‐aqueous interface, which remains independent of the adsorption and desorption dynamics of Glu and Asp. Additionally, we observe no transition from its tangential configuration (Figure 4D) upon temperature changes, with *Γ*_m_ remaining nearly identical to the *Γ*_c_ values calculated using the temperature‐dependent Δ*n* of E7 (Figure S7B).

Combined with the DFT results suggesting the energetic transition of LC orientation upon bindings with Glu and Asp, therefore, these experiments demonstrate that the anchoring transition triggered by the interfacial adsorption of Glu and Asp is associated not with the formation of isotropic LC domains (mechanism i) but with the change in the easy axis of interfacial LCs in favor of the tangential anchoring (mechanism ii). We note that, as shown in Figure S8, the thin homeotropic LC films (*d* = 3.6 µm, no **OI‐C12**, Figure 2A) also show the consistent results with the thick LC films (Figure 4), confirming that the presence of **OI‐C12** does not preclude the interfacial behavior of Glu and Asp at the LC‐aqueous interface.

### 2.4 Interfacial Dynamics of Glu/Asp upon their Complex Formation with *Salmonella* Lipopolysaccharide (S‐LPS)

**FIGURE 5:**
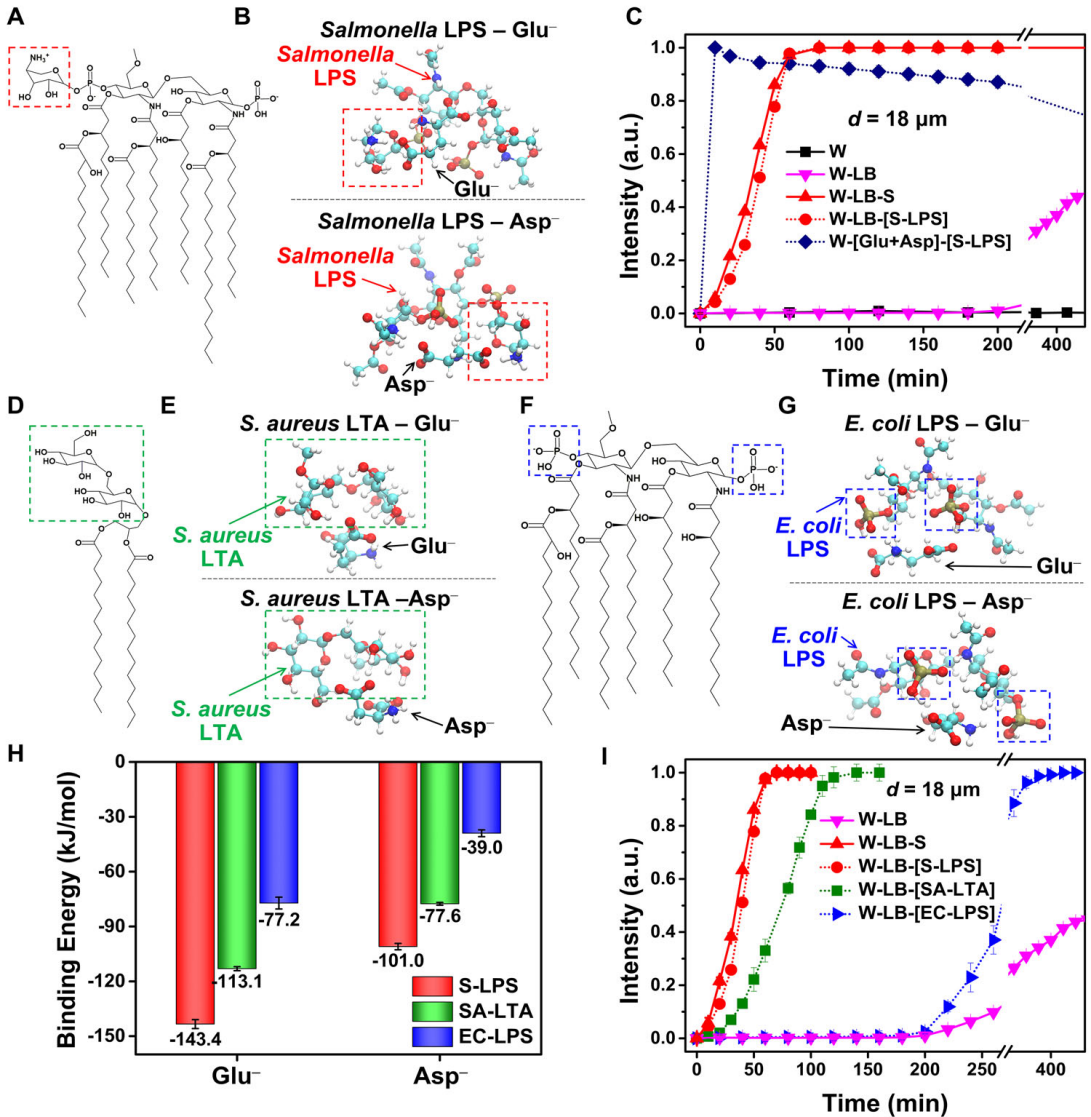
Complex formation of Glu^–^/Asp^–^ with bacterial byproducts and resulting optical responses of the LC films. (A) Molecular structure of *salmonella* lipid A (a significant component of *salmonella* lipopolysaccharide, S‐LPS) and (B) its lowest‐energy DFT‐optimized binding geometries with Glu^–^/Asp^–^. Red boxes represent 4‐amino‐4‐deoxy‐L‐arabinose (Ara4N) in S‐LPS. (C) Intensity profiles of the thick LC films (*d* = 18 µm, with **OI‐C12**) in the W‐LB‐[S‐LPS] and W‐[Glu+Asp‐S‐LPS] solutions compared with those in the pure water, W‐LB, and W‐LB‐S solutions. (D) Molecular structure of *Staphylococcus aureus* (*S. aureus*) lipoteichoic acid (SA‐LTA) and (E) its lowest‐energy DFT‐optimized binding geometries with Glu^–^/Asp^–^. Green boxes represent lactose in SA‐LTA. (F) Molecular structure of *Escherichia coli* (*E. coli*) LPS (EC‐LPS) and (G) its lowest‐energy DFT‐optimized binding geometries with Glu^–^/Asp^–^. Blue boxes represent phosphate groups in EC‐LPS. (H) Ensemble‐averaged DFT‐calculated average binding energies (kJ/mol) for Glu^–^/Asp^–^ with S‐LPS, SA‐LTA, and EC‐LPS, evaluated over the ten lowest‐energy DFT‐refined minima and Boltzmann‐weighted at *T* = 300 K; error bars indicate the corresponding Boltzmann‐weighted standard deviation across these minima (structure/pose‐dependent variability). The individual binding energies for the ten lowest‐energy DFT‐refined minima are shown in Figure S9. (I) Intensity profiles of the thick LC films (*d* = 18 µm, with **OI‐C12**) in the W‐LB solutions with S‐LPS, SA‐LTA, and EC‐LPS.

Building from our deep understanding on the intermolecular interactions between LCs and the amino acids of Glu/Asp, we now explore why the presence of *Salmonella* in the W‐LB solution significantly accelerates the anchoring and optical transitions of LC films (see red line in Figure 1G). As a key potential determinant for the rapid transition, we focus on the bacterial byproduct, lipopolysaccharide (LPS) that is the major cell‐wall component of Gram‐negative bacteria (e.g., *Salmonella*, *Escherichia coli* (*E. coli*)) [59]. Past studies have verified that a specific part of LPS, lipid A (Figure 5A), drives the formation of LPS‐peptide complexes through electrostatic and hydrophobic interactions [60, 61, 62]. Therefore, we deduce that the *Salmonella* LPS (S‐LPS) similarly interacts with anionic Glu^–^ and Asp^–^ to form stable S‐LPS complexes. To validate this hypothesis at the molecular level, we perform DFT calculations on S‐LPS–Glu^−^/Asp^−^ complexes. Given the conformational flexibility—particularly torsional variability around glycosidic linkers—and its heterogeneous charge distribution, we employ an ensemble‐based workflow (see Experimental Section): conformer/rotamer sampling generated candidate structures, the ten lowest‐energy candidates are refined at the DFT level (Figure S9A,D), and the lowest‐energy optimized binding geometry is reported. As expected, our DFT‐optimized geometries (Figure 5B) reveal that the negatively charged carboxylate of Glu^–^ preferentially coordinates with the positively charged ammonium and hydroxyl moieties of the 4‐amino‐4‐deoxy‐L‐arabinose (Ara4N) group in S‐LPS (red boxes in Figure 5A,B). A similar coordination pattern is observed for Asp^–^ binding to S‑LPS. To quantitatively assess the physical origin of this stabilization, we perform fragment‐pairwise local energy decomposition (fp‐LED) analysis [63], which decomposes the inter‐fragment interaction energy between predefined molecular fragments (Figure S10A) into physically meaningful components [64, 65]. The fp‐LED total inter‐fragment interaction maps for the S‐LPS–Glu^−^ and S‐LPS–Asp^−^ complexes (Figure S10B,C) show that the dominant stabilizing contributions are concentrated in a small number of fragment pairs between Glu^−^/Asp^−^ and the S‐LPS headgroup. To elucidate the physical origin of these key contacts, we further decompose each fragment‐pair interaction into component terms—electronic preparation (Δ*E*_el‐prep_), electrostatics (*E*_elec_), dielectric (*E*_diel_), exchange (*E*_exch_), dispersion (*E*_disp_), and non‐dispersion (*E*_non‐disp_) contributions; Figure S10D,E). This component‐resolved analysis reveals that while substantial electronic preparation is required to polarize/relax the fragments upon complex formation (Δ*E*_el‐prep_), the net stabilization of the dominant fragment pairs is governed primarily by electrostatics with dielectric screening (*E*_elec_+*E*_diel_), particularly for interactions involving the Ara4N ammonium‐bearing fragment, whereas dispersion and other terms provide secondary contributions. Notably, for Asp^−^, additional stabilizing interactions are also observed for fragment pairs involving the phosphate‐containing fragment, consistent with a specific P–OH···O^−^ hydrogen bond accompanied by electrostatic stabilization (Figure S10E). Although Asp^–^ can form a stabilizing hydrogen bond between a carboxylate oxygen and a phosphate hydroxyl group, this gain is largely offset by strong repulsive interactions involving the negatively charged phosphate groups in S‐LPS, leading to weaker overall stabilization for Asp^–^ than for Glu^–^. This is consistent with the favorable binding energies of −143.4 kJ/mol for Glu^–^ and −101.0 kJ/mol for Asp^–^ (red bars in Figure 5H).

On the basis of these theoretical predictions, we examine the temporal intensity evolution of the thick homeotropic LC films (*d* = 18 µm, with **OI‐C12**) in the W‐LB solution with S‐LPS (W‐LB‐[S‐LPS], dotted‐red line in Figure 5C) and in the W‐[Glu+Asp]‐[S‐LPS] solution (dotted‐navy line in Figure 5C), and compare them with that in the W, W‐LB, and W‐LB‐S solutions investigated above (Figure 1G and also shown in 5C as solid lines); the concentration of S‐LPS in the solutions is 5 µg/ml. From the measured intensity profiles in Figure 5C, we can make three key observations.

First, analogous to the result in the W‐LB‐S solution (solid‐red line in Figure 5C), we observe the presence of S‐LPS in the W‐LB solution to also significantly accelerate the dark‐to‐bright optical transition (dotted‐red line in Figure 5C) as compared to that in the W‐LB solution (solid‐pink line in Figure 5C). In addition, the intensity profile in the W‐LB‐[S‐LPS] solution is comparable with that in the W‐LB‐S solution (see solid‐ and dotted‐red lines in Figure 5C), proving that S‐LPS from *SSalmonella* cells is the key component to trigger the rapid anchoring/optical transition of LCs. Second, as demonstrated by past works [15], the greater number of aliphatic tails in biomolecules (e.g., LPS, endotoxin) profoundly promotes not only their self‐assembly at the LC‐aqueous interface but also their orientational couplings with LCs. Therefore, the S‐LPS complexes with Glu/Asp (seven tails, Figure 5A,B) should enforce the adsorption and stabilization of Glu^–^ and Asp^–^ at the LC‐aqueous interface more than the Lys complexes (single tail, Figure 2G). This is responsible for the faster anchoring/optical transition of the LC films in the W‐LB‐S solution, where the S‐LPS complexes form (solid‐red line in Figure 5C), than in the W‐LB solution producing the Lys complexes (solid‐pink line in Figure 5C). Third, in Figures 2, 3, 4, three compositions in the LB broth (Glu, Asp, and Lys) are determined to play a major role in triggering the anchoring transition of LCs. We observe that S‐LPS can even more accelerate the optical transition of LC films in the absence of Lys (i.e., in the W‐[Glu+Asp]‐[S‐LPS] solution, dotted‐navy line in Figure 5C) than in the presence of Lys (i.e., W‐LB‐[S‐LPS] solution, dotted‐red line in Figure 5C). This result hints that some fractions of Glu and Asp in the W‐LB solution already bind with Lys before the introduction of *salmonella* (i.e., S‐LPS) and thus cannot form the complexes with S‐LPS. The existence of certain portions of Lys complexes, therefore, slows down the adsorption of Glu and Asp at the LC‐aqueous interfaces, which is in line with our conclusion from the second observation regarding the enhanced interfacial adsorption of Glu/Asp complexes with S‐LPS than with Lys. To verify that S‐LPS–Glu^−^ complex also retains (and potentially amplifies) the orientation preference at the LC interface, we additionally compute its binding to 5CB in two representative arrangements: tangential (face‐to‐face) and homeotropic (edge‐to‐edge) configurations (Figure S11). In the face‐to‐face configuration, the optimized geometries reveal that the primary Glu^−^–5CB interaction motif is largely preserved. In addition, the ammonium group in the S‐LPS headgroup can approach the aromatic core of 5CB, providing an extra stabilization via strong cation–π interaction (Figure S11A). By contrast, in the edge‐to‐edge configuration, the amino‐acid ammonium group that contributes to stabilizing the homeotropic Glu^−^–5CB arrangement becomes engaged in strong intra‐complex interactions within S‐LPS–Glu^−^, reducing its availability for favorable interaction with 5CB and thereby perturbing the preferred homeotropic binding motif (Figure S11B). Consequently, S‐LPS complexation strengthens the face‐to‐face binding while weakening the edge‐to‐edge binding, increasing the face‐over‐edge preference from ∆*E* = −7.3 kJ/mol (Glu^−^) to ∆*E* = −36.6 kJ/mol (S‐LPS–Glu^−^) (Figure S11C). Together, these results indicate that LPS–Glu^−^ complexation reshapes the LC–analyte interaction landscape and can enhance orientational coupling. We note that our DFT model does not include the full lipid environment; in the complete amphiphilic LPS architecture, hydrophobic acyl chains are expected to further promote association with the LC film via interdigitation, which could strengthen interfacial anchoring and coupling beyond what is captured here.

To provide further insight into the electrostatic interaction of bacterial byproducts with the amino acids of Glu/Asp and their orientational coupling with LCs, we explore two additional byproducts from other food poisoning bacteria that possess differently charged moieties, lipoteichoic acid (LTA) from *Staphylococcus aureus* (*S. aureus*) (SA‐LTA, Figure 5D) and LPS from *E. coli* (EC‐LPS, Figure 5F). We note that LTA is a cell‐wall component of Gram‐positive bacteria (e.g., *S. aureus*) and is also known to form complexes with peptides, like the LPS in Gram‐negative bacteria (e.g., *Salmonella*, *E. coli*) [66]. Because the cationic moieties, which is present in S‐LPS, are omitted in SA‐LTA and EC‐LPS, our DFT calculations reveal that the bindings of both Glu^–^ and Asp^–^ with SA‐LTA (Figure 5E) and EC‐LPS (Figure 5G) are energetically less favorable than with S‐LPS (Figure 5B). Nevertheless, the fp‐LED results indicate that favorable interactions still exist—primarily between the amino acid carboxylate and hydroxyl‐bearing motifs in SA‐LTA (Figure S12), and phosphate‐containing motifs in EC‐LPS (Figure S13)—although these contributions are much weaker than the dominant carboxylate–ammonium electrostatic interaction observed for S‐LPS (Figure S10). As plotted in Figure 5H, the ensemble‐averaged binding energies of Glu^–^ and Asp^–^ with the bacterial byproducts over the ten lowest‐energy candidates (Figure S9B,E for SA‐LTA; Figure S9C,F for EC‐LPS) are ranked as |*E*(S‐LPS)| > |*E*(SA‐LTA)| > |*E*(EC‐LPS)|. These theoretical calculations are in line with our experimental results (Figure 5I) showing the fastest optical transition of LC films in the W‐LB solution with S‐LPS, followed by SA‐LTA and EC‐LPS; the concentration of bacterial byproducts in the solutions is 5 µg/ml. It is worth pointing out that the optical transition of LC films in the W‐LB‐[SA‐LTA] is faster than that in the W‐LB‐[EC‐LPS] despite the smaller number of aliphatic tails in SA‐LTA (two tails) than EC‐LPS (six tails). This indicates that the intermolecular interaction between the bacterial byproducts and amino acids is critical for triggering the anchoring transition of LCs because the formation of complexes with amphiphilic bacterial byproducts stabilizes this adsorbed state by hindering desorption and increasing both the interfacial residence time and the surface coverage of amino acids that trigger tangential anchoring. Collectively, the anchoring and optical transitions likely reflect a combination of (i) the tendency to form complexes (captured by the binding strength, which controls the concentration and surface coverage of complexes) and (ii) the hydrophobic coupling of the complexes with LCs (influenced by the number and length of aliphatic tails). The faster response of LCs with SA‐LTA complexes than EC‐LPS complexes suggests that stronger complex formation (and the resulting higher complex concentration and surface coverage) can preclude differences in tail number, while S‐LPS dominates in both strong complex formation and a larger number of tails. The correlation between *E* and the anchoring/optical transition of LCs offers the basic principle for designing the LC system to identify a specific biomolecular interaction, which can be extended in practical applications, including biosensor, medical diagnostics, and drug screening [14, 15, 16].

### 2.5 Extraordinary Sensitivity and Response Time of the LC‐Based Recognition System

**FIGURE 6:**
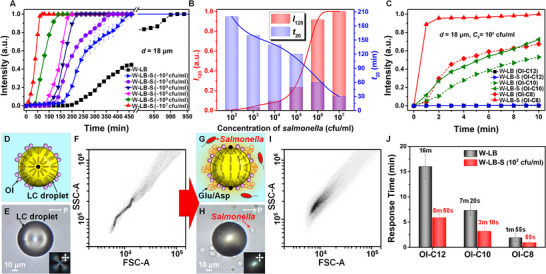
Optical response of the LC system dependent on *C*_S_, aliphatic tail length of **OI**, and LC geometry. (A) *C_S_*‐dependent optical response of the thick homeotropic LC film (*d* = 18 µm, with **OI‐C12**) in the W‐LB‐S solutions. (B) Corresponding optical intensities at 120 min incubation (*I*_120_, red bars and line) and response times at 20% of maximum optical intensity (*t*_20_, blue bars and line) as a function of the *C_S_*. (C) Tail length‐dependent optical responses of the thick homeotropic LC films (*d* = 18 µm) treated with **OI** having aliphatic tails of twelve (**OI‐C12**), ten (**OI‐C10**), or eight carbon atoms (**OI‐C8**) in the W‐LB and W‐LB‐S solutions. *C*_LB_ = 16.7% (v/v) and *C_S_* ∼ 10^2^ cfu/ml. (D–I) (D, G) Schematic illustrations and (E, H) corresponding micrographs and (F, I) flow cytometry patterns for the LC droplets treated with **OI‐C12** in (D‐F) the pure water and (G‐I) W‐LB‐S (*C*_LB_ = 16.7% (v/v) and *C_S_* ∼ 10^2^ cfu/ml) solutions. SSC and FSC in (F) and (I) indicate side scatter and forward scatter, respectively. LC droplets with the internal LC ordering in (D) and (G) are referred to as the radial and the bipolar configurations, respectively. The black dots in (D) and (G) represent topological defects. (J) Radial‐to‐bipolar transition times of the LCs droplets treated with **OI‐C12**, **OI‐C10**, and **OI‐C8** in the W‐LB and W‐LB‐S solutions. *C*_LB_ = 16.7% (v/v) and *C_S_* ∼ 10^2^ cfu/ml.

Based on the working principles regarding the significant orientational coupling of LCs with the amino acids of Glu/Asp and their complexes with S‐LPS, we investigate the optical/anchoring transition characteristic of LCs with respect to (i) *C_S_*, (ii) aliphatic tail length of **OI**, and (iii) LC geometry to enable access to the LC system with high level of control over sensitivity and response time, which is critical to realize its full potential for practical applications. First, the optical signal of LCs is expected to strongly depend on *C_S_* because the higher *C_S_* would drive the formation of the larger number of S‐LPS complexes with Glu/Asp and thus promote their adsorption at the LC‐aqueous interface. To evaluate the effect of *C_S_* on the anchoring transition of LCs, we prepare the W‐LB‐S solutions with different *C_S_* obtained by culturing *Salmonella* cells of 10 cfu/ml with varying culturing time (*t*_c_) at typical culturing *T* = 37°C. The prepared *C_S_* are 10^2^ cfu/ml (*t_c_* = 0.5 h), 10^3^ cfu/ml (*t*_c_ = 1 h), 10^4^ cfu/ml (*t*_c_ = 2 h), 10^5^ cfu/ml (*t*_c_ = 3 h), 10^6^ cfu/ml (*t*_c_ = 4 h), and 10^7^ cfu/ml (*t*_c_ = 4.5 h). In the W‐LB‐S solution with higher *C_S_*, the thick homeotropic LC films (*d* = 18 µm, with **OI‐C12**) show faster optical responses (Figure 6A) as expected, leading to the correlation between *C_S_* and the optical intensity of LCs which can be written as

(1) $$\begin{array}{ccc} I_{120} & = & -6.0\cdot 10^{-2}\cdot \exp\left(-\frac{C_{S}}{1.1\cdot 10^{3}}\right)\\ & & -9.4\cdot 10^{-1}\cdot \exp\left(-\frac{C_{S}}{4.2\cdot 10^{5}}\right)+1 \end{array}$$

where *I*_120_ is the optical intensity of the LC film at 120 min incubation (red bars and line in Figure 6B). In addition, we also evaluate the response time (*t*_20_, blue bars and line in Figure 6B) at which the LC film exhibits 20% of its maximum intensity with respect to *C_S_*, which can be described as

(2) $$\begin{array}{ccc} t_{120} & = & 1.1\cdot {C_{S}}^{4}-2.1\cdot 10^{1}\cdot {C_{S}}^{3}+1.4\cdot 10^{2}\cdot {C_{S}}^{2}\\ & & -4.1\cdot 10^{2}\cdot C_{S}+6.1\cdot 10^{2} \end{array}$$

This result implies that the LC system allows one to not only optically recognize the presence of *Salmonella* cells and their biomolecular interactions with amino acids but also precisely quantify *C_S_* through the above equations.

Second, in order to control the sensitivity of LC film, we modulate the stability of initial homeotropic LC anchoring at the LC‐aqueous interface by altering the aliphatic tail length in the **OI** (which is responsible for the initial homeotropic LC configuration [33]) from twelve carbons in alkyl chain of **OI‐C12** to ten (**OI‐C10**, Figure S14A) and eight carbons (**OI‐C8**, Figure S14B). When incubated in the W‐LB solution (dotted‐black lines in Figure 6A,C) or the W‐LB‐S solution with *C_S_* ∼ 10^2^ cfu/ml (solid‐blue lines in Figure 6A,C), the thick LC films (*d* = 18 µm, with **OI‐C12**) show no sign of optical responses for long period of incubation time over 150 min because the long aliphatic tails of **OI‐C12** interdigitated between LC molecules promote a preference for the homeotropic anchoring of LCs, resulting in high tolerance for the homeotropic‐to‐tangential anchoring transition triggered by adsorbates [33]. When **OI‐C12** is replaced by **OI‐C10** or **OI‐C8**, however, we observe significantly faster and more intense optical responses from the LC films in both the W‐LB solution (dotted‐green and ‐red lines in Figure 6C) and the W‐LB‐S solution with *C_S_* ∼ 10^2^ cfu/ml (solid‐green and ‐red lines in Figure 6C) due to a reduction in the LC‐**OI** orientational coupling by the aliphatic tails. Importantly, the thick LC films with **OI‐C10** or **OI‐C8** in the W‐LB‐S solutions exhibit notably distinguishable optical signals from those in the W‐LB solutions. Therefore, these results provide simple and versatile principles for designing the LC systems that are capable of recognizing and optically reporting the presence of *S = Salmonella* cells even at the trace concentration level of *C_S_* ∼ 10^2^ cfu/ml (10% level of infective dose) with the extremely reduced recognition time of less than 1 min (solid‐red line in Figure 6C) as compared to the conventional methods (e.g., PCR and ELISA) requiring the assay time of at least 6 h [29].

Third, past studies showed that a droplet geometry induces a large elastic strain of LCs, thus promoting more sensitive anchoring/optical transitions in response to interfacial adsorption of analytes [15, 67]. In addition, since LC droplets show distinct light scattering patterns depending on the internal LC ordering, the droplet geometry allows for the use of flow cytometry that can rapidly analyze large population of droplets (up to 10 000 droplets per min) and thereby offer robust statistic results [34, 67]. Specifically, the homeotropic and tangential anchoring conditions lead to distinct internal ordering in the LC droplets to meet the geometrical theorem [68], so‐called the radial configuration with a topological defect located at the center of droplet (Figure 6D) and the bipolar configuration with two topological defects at the poles (Figure 6G), respectively. We also observe the LC droplets with **OI‐C12** to assume the homeotropic anchoring, evidenced by the POM texture corresponding to the radial configuration (Figure 6D,E; Figure S15A,B). When examined under the flow cytometry (with a flow rate of 14 µl /min and ∼30 000 LC droplets), the homeotropic droplets exhibit S‐shape pattern (Figure 6F) in the intensity plot of side scattered light scattering (SSC) against forward light scattering (FSC), which has been shown to be the characteristic scattering pattern from the radial configuration [34, 67]. In the W‐LB‐S solution (*C_S_* ∼ 10^2^ cfu/ml), however, we observe that all LC droplets undergo the rapid ordering transition from the radial to bipolar configurations (i.e., homeotropic‐to‐tangential anchoring transition) within 6 min (Figure 6G,H,J; Figure S15C,D), resulting in the corresponding broad light scattering pattern (Figure 6I) [34, 67]. Furthermore, consistent with the results in LC films (Figure 6C), the LC droplets treated with **OI‐C10** and **OI‐C8** show faster responses (3 min and 1 min, respectively) in the W‐LB‐S solution of *C_S_* ∼ 10^2^ cfu/ml (Figure 6J). We point out three important features from the droplet geometry: (i) While the LC films with **OI‐C12** in the W‐LB‐S solution (*C_S_* ∼ 10^2^ cfu/ml) generate no optical signal over 2 h (solid‐blue line in Figure 6A) and also require more than 7 h to reach the maximum intensity (i.e., full anchoring/optical transition), the LC droplets can make the full transition only within 6 min (red bars in Figure 6J), as demonstrated by the distinct POM texture and light scattering pattern (Figure 6D–I); (ii) When treated with **OI‐C10**, the response time of LC droplets for the full transition further decreases to ∼ 3 min (red bars in Figure 6J) at which the LC films are still in the middle of anchoring transition displaying bright domains (like Figure 1D) and only ∼ 35% of maximum intensity (solid‐green line in Figure 6C); (iii) Although the anchoring transition of LCs in the W‐LB solutions is also dramatically accelerated in the droplet geometry (black bars in Figure 6J), the response times of LC droplets in the W‐LB‐S solution (red bars in Figure 6J) are still distinct enough to be discriminated. Collectively, these results provide unprecedented opportunities for designing the LC system to autonomously recognize and optically report a specific biological interaction between bacterial LPS and amino acids, with extraordinary sensitivity and recognition time.

## 3 Conclusions

In summary, on the basis of our findings regarding the significant orientational coupling of LCs with the amino acids and their biomolecular complexes, we design the LC‐based optical transducer to recognize the specific interfacial behavior of biomolecules and report it through the distinct macroscopic optical signal. We demonstrate two specific amino acids of Glu and Asp to exhibit the unique adsorption‐desorption dynamics at the LC‐aqueous interfaces to cause the transient orientational transition of interfacial LCs from the initial homeotropic to tangential anchoring, accompanied by the alternating optical signals of LCs between the dark (homeotropic anchoring) and bright states (tangential anchoring). In this process, another biomolecule, Lys, is shown to interact with Glu and Asp to form Lys complexes that promote the adsorption of Glu/Asp at the LC interfaces but hinder their desorption, leading to the distinct optical signals of accelerated dark‐to‐bright optical transition and persistent bright state. In addition, we verify the interfacial behavior of Glu/Asp and their interplay with LCs to be governed by pH condition of the aqueous solutions because of significant changes in the charge states of amino acids. From the theoretical estimation for pH‐dependent dissociation fraction of Glu and Asp, net charge of which becomes more negative at higher pH, we experimentally verify that (i) the negatively charged Glu^–^ and Asp^–^ are more preferentially adsorbed and stabilized at the LC‐aqueous interface than other species, (ii) the neutrally charged Glu^0^ and Asp^0^ are responsible for the transient anchoring transition (i.e., desorption of Glu and Asp), and (iii) the interfacial adsorption of positively charged Asp^+^ is significantly stronger than that of Glu^+^. In good agreement with our experimental results, DFT calculations validate that the anionic Glu^–^/Asp^–^, neutral Glu^0^/Asp^0^, and cationic Asp^+^ energetically favor to interact with LCs in the face‐to‐face binding configuration to orient the LC molecules tangentially at the LC‐aqueous interfaces (which is responsible for the dark‐to‐bright optical transition), while the cationic Glu^+^ tends to induce the edge‐to‐edge configuration corresponding to the homeotropic anchoring of LCs (i.e., no or weak optical transition from the initial dark state). Furthermore, we uncover dramatic acceleration of the orientational/optical transition of LCs in the presence of *Salmonella* cells along with Glu and Asp to be associated with a specific interaction between S‐LPS and anionic Glu^–^ and Asp^–^, leading to the formation of S‐LPS complexes. As compared to the Lys complex possessing a single aliphatic tail, the LPS complex has a larger number of aliphatic tails (seven tails), which prefer to interdigitate between LC molecules, thereby further enforcing the adsorption and stabilization of Glu^–^ and Asp^–^ at the LC‐aqueous interface (i.e., higher interfacial residence time and coverage). Other types of bacterial byproducts (SA‐LTA and EC‐LPS) are also verified to form complexes with Glu/Asp that are recognized by the LCs with distinct optical transition rates. Conformer/rotamer ensemble sampling followed by DFT refinement identifies specific binding motifs and indicates that S‐LPS‐Glu^−^/Asp^−^ complexes bind more strongly than the corresponding SA‐LTA and EC‐LPS complexes. Moreover, LED analysis attributes the stabilization primarily to favorable electrostatic interaction between the amino acid carboxylate and the Ara4N ammonium‐containing motif, thereby providing a physically interpretable link between molecular interactions and the experimentally observed trends in LC responses. Finally, building from additional investigations on the recognition characteristics of LC systems with respect to *C_S_*, aliphatic tail length of **OI**, and LC geometry, we provide access to high level of control over the sensitivity and recognition time. As a result, by adopting the **OI** with shorter aliphatic tails and the droplet geometry, we realize the practical potential of LC system to recognize the trace concentration level of *Salmonella* cells (10^2^ cfu/ml) with the recognition time of less than 1 min. Looking forward, explicit‐interface molecular dynamics simulations—ideally coupled with free‐energy methodologies—will enable a more quantitative connection between interfacial adsorption/orientation bias and interdigitation‐driven interfacial restructuring to measurable orientational/optical transitions, thereby guiding predictive design rules. We envisage that the comprehensive understanding on the underlying mechanisms described in this study will not only provide fundamental insights into interfacial dynamics of biomolecules and their interactions with other biological species but also hint at the practical potential of LC‐based recognition systems in a wide variety of fields including biological sensors, medical diagnostics, and environmental monitoring.

## 4 Experimental Section

### 4.1 Materials

The liquid crystal (LC, E7) was purchased from Jingsu Hecheng Display Technology Co., Ltd. (China). N,N‐dimethyl‐N‐octadecyl‐3‐aminopropyltrimethoxysilyl chloride (DMOAP, 42 wt.% in methanol), Luria‐Bertani (LB) broth (Miller), Glutamic acid (Glu), Aspartic acid (Asp), *salmonella* lipopolysaccharides (LPS), and photoresist and developer for preparing 3.6 µm‐thick grids (AZ LOR‐28 and AZ300 MIF Developer, respectively) were purchased from Merck (Germany). 2‐Propanol (99.9%) was purchased from Samchun Chemical Co., Ltd. (Republic of Korea). pH buffer solutions (pH 1.68, pH 4.01, pH 7.00, pH 10.01) were purchased from Thermo Fisher Scientific (United States). Bare glass for substrate was purchased from Zhuhai Kaivo Optoelectronic Technology Co., Ltd. (China). 18 µm‐thick copper grids for transmission electron microscopy (TEM) were purchased from Electron Microscopy Sciences (United States). All chemicals and solvents were analytical grade and used as received. Organic ionic plastic crystal (**OI**) was synthesized in our laboratory.

### 4.2 Preparation of DMOAP‐Coated Glass

The glass substrates were submerged in a 3% (v/v) DMOAP solution, followed by bath sonication for 10 min. Subsequently, the substrates were rinsed with deionized (DI) water and ethanol.

### 4.3 Preparation of 3.6 µm‐Thick Microwell using AZ LOR‐28

Microwells with a thickness of 3.6 µm were prepared on a glass substrate using AZ LOR‐28 photoresist. First, the AZ LOR‐28 solution was spin‐coated onto the glass substrate at 3500 rpm for 30 s. The coated substrate was then soft‐baked at 100°C for 300 s to remove residual solvents. The resulting thickness of AZ LOR‐28 film was measured to be 3.6 µm. For patterning the microwells, the sample was exposed to ultraviolet light for 30 s through a photomask designed with microwell arrays, followed by a post‐exposure back at 110°C for 300 s. Subsequently, the microwells were developed in a developer (AZ300 MIF Developer) for 40 s. Finally, the microwells were washed three times using 2‐Propanol and DI water.

### 4.4 Preparation of LC Films

An 18 µm‐thick copper grid (for TEM) or a 3.6 µm‐thick microwell array prepared using AZ LOR‐28 was placed onto the DMOAP‐coated substrate to prepare microwells, which were then filled with a mixture of nematic LC (E7) and **OI**. The **OI** was used to achieve homeotropic of LCs at the LC‐aqueous interface. The concentration of the **OI**s doped into the LC phase—specifically **OI‐C12, OI‐C10**, and **OI‐C8**, which possesses twelve, ten, and eight carbons in their aliphatic tails, respectively—was 0.2 mM, 0.7 mM, 1 mM.

### 4.5 Preparation of LC Droplets

LC droplets were formed by vortex mixing of 1% (v/v) of the mixture of a nematic LC (E7) and **OI** in DI water at 3000 rpm for 5 s. The **OI** was used to achieve homeotropic of LCs at the LC‐aqueous interface, leading to radial configuration in LC droplets. The concentrations of **OI‐C12**, **OI‐C10**, **OI‐C8** in the LC droplets were 1 mM, 1.5 mM, 2.5 mM, respectively.

### 4.6 Culturing Protocol for Bacterial Cells

*C_S_* ∼ 10 cfu/ml of *salmonella* was cultured in LB broth at 37°C over 4.5 h with the time intervals of 0.5 h (culturing time, *t_c_* = 0.5 h for ∼ 10^2^ cfu/ml, *t_c_* = 1 h for ∼ 10^3^ cfu/ml, *t_c_* = 2 h for ∼ 10^4^ cfu/ml, *t_c_* = 3 h for ∼ 10^5^ cfu/ml, *t_c_* = 4 h for ∼ 10^6^ cfu/ml, *t_c_* = 4.5 h for ∼ 10^7^ cfu/ml). The concentrations of bacterial solutions were determined by cell count.

### 4.7 Preparation of *Salmonella* Dispersed Water

*Salmonella* dispersed in LB broth was centrifuged at 4000 rpm for 5 min, and then the supernatant liquid was removed. The remaining *salmonella* was diluted with DI water. This procedure was repeated for 5 times to minimize the influence of remaining LB broth.

### 4.8 Calculation of the Fraction of Dissociation for Lys in Pure Water (pH 6.9)

The fraction of dissociation for polyprotic acid for Lys ($\alpha_{H_{3}A^{+}}$ for positively charged Lys^+^, $\alpha_{H_{2}A}$ for neutrally charged Lys^0^, and $\alpha_{HA^{-}}$ for negatively charged Lys^–^) can be described as

(3) $$\alpha_{H_{3}A^{+}}=\frac{\left[H_{3}A^{+}\right]}{\left[F\right]}=\frac{{\left[H^{+}\right]}^{2}}{\left[F\right]}\;$$

(4) $$\alpha_{H_{2}A}=\frac{\left[H_{2}A\right]}{\left[F\right]}=\frac{\left[H^{+}\right]\cdot K_{a1}}{\left[F\right]}\;$$

(5) $$\alpha_{HA^{-}}=\frac{\left[HA^{-}\right]}{\left[F\right]}=\frac{K_{a1}\cdot K_{a2}}{\left[F\right]}\;$$

where[*F*]  =  [*H* ^+^]^2^ + [*H* ^+^] · *K*_*a*1_ + *K*_*a*1_ · *K*_*a*2_, *K*_a1_ and *K*_a2_ are the equilibrium constants of the acids (10^−2.18^ and 10^−8.95^ for Lys, respectively), and [H^+^] denotes the molar concentration of H^+^ [50]. According to these equations, we calculated the fraction of dissociations for Lys in pure water (pH 6.9). The results reveal that positively charged Lys^+^ (99.12%, Figure 2G) are dominantly present and followed by neutrally charged Lys^0^ (0.88%). Therefore, Lys in pure water denotes Lys^+^ in this study.

### 4.9 Calculation of the Fraction of Dissociations for Glu and Asp in Aqueous Solutions at pH 1.5, pH 3.6, and pH 6.2

When Glu or Asp (*C*_Glu_ = 3.2 mM or *C*_Asp_ = 1.2 mM, which is equivalent with that in the W‐LB solution of *C*_LB_ = 16.7% (v/v)) is introduced into pH buffers with the initial pH values of 4, 7, and 10, the equilibrium pH reaches 1.5, 3.6, and 6.2, respectively. According to Equations (3), (4), (5), we calculated the fraction of dissociation for polyprotic acid, Glu, and Asp in aqueous solutions at pH 1.5 ($\alpha_{H_{3}A^{+}}$ for positively charged Glu^+^/Asp^+^, $\alpha_{H_{2}A}$ for neutrally charged Glu^0^/Asp^0^, and $\alpha_{HA^{-}}$ for negatively charged Glu^–^/Asp^–^), where *K*_a1_/*K*_a2_ are 10^−2.19^/10^−4.25^ for Glu and 10^−1.88^/10^−3.65^ for Asp, respectively. The calculated fraction of dissociations for Glu are 80.98% for Glu^+^, 18.98% for Glu^0^, and 0.04% for Glu^−^ and that for Asp are 70.43% for Asp^+^, 29.36% for Asp^0^, and 0.21% for Asp^–^ (Figure 3C). At pH 3.6 and pH 6.2 where pH is higher than the isoelectric point (*pI* = 3.22 for Glu and 2.77 for Asp), however, the relation between the pH of acids in aqueous solutions and their *pK*_a_ (acid dissociation constant) can be written as $pH=pK_{a}+\log\frac{\left[A^{-}\right]}{\left[HA\right]}$, which is called Henderson‐Hasselbalch equation, where [A^–^] and [HA] denote the molar concentration of the conjugate base of the acid (Glu^–^/Asp^–^ at pH 3.6 and Glu^2–^/Asp^2–^ at pH 6.2) and the weak acid (Glu^0^/Asp^0^ at pH 3.6 and Glu^–^/Asp^–^ at pH 6.2), respectively. For Glu/Asp, the values of *pK*_a1_, *pK*_a2_, and *pK*_a3_ are 2.19/1.88, 4.25/3.65, and 9.67/9.60, respectively. According to Henderson‐Hasselbalch equation, we calculated the fraction of dissociations for Glu and Asp in the aqueous solutions at pH 3.6 (82.6%/17.4%/51.3%/48.7% for Glu/Glu^−^/Asp/Asp^−^) and pH 6.2 (99.97%/0.03%/99.95%/0.05% for Glu^–^/Glu^2–^/Asp^–^/Asp^2–^, Figure 3C).

### 4.10 Calculation of Optical Retardance

The optical retardance (*Γ*_c_) of LC films under hybrid anchoring conditions can be described as $\Gamma_{c}=\frac{|n_{e}-n_{o}|\cdot d}{2}$ [13, 58], where *n*_e_ and *n*_o_ are the extraordinary and ordinary refractive indices, and *d* is the thickness of the LC films. For E7, the optical birefringence is positive, *n*_e_ − *n*_o_ ≈ 0.224. According to this equation, we calculated the *Γ*_c_ = 403 nm for *d* = 3.6 µm and 2016 nm for *d* = 18 µm.

### 4.11 DFT Calculations

All DFT calculations were carried out with the Gaussian 16 software package [69] at the B3LYP‐GD3BJ/6‐31+G(d,p) level of theory [70, 71, 72, 73, 74]. Solvent effects were modeled using Tomasi's polarizable continuum model with the self‐consistent reaction field (PCM‐SCRF) [75, 76], employing water as the solvent. Molecular geometries were first optimized via the Berny algorithm [77], followed by single‐point calculations to obtain the total electronic energies. The basis set superposition error (BSSE) was corrected using the Boys and Bernardi counterpoise (CP) method [78]. We note that, owing to their nonpolar character, the hydrophobic tails are not expected to significantly affect the calculated binding energies. Although truncating these tails reduces conformational complexity, the binding between anionic amino acids and sugar‐rich LPS/LTA headgroups can remain highly sensitive to internal rotamers and local binding poses. To account for this, we introduced a more systematic approach for structural exploration (see Conformational sampling).

### 4.12 Conformational Sampling

We employed a hierarchical sampling‐refinement protocol and quantified the resulting structure/pose‐dependent variability across low‐energy minima. Starting from a DFT‐optimized reference structure for each system (S‐LPS, SA‐LTA, EC‐LPS, and their complexes with Glu^–^/Asp^–^), we performed automated intermolecular pose exploration and internal conformer/rotamer sampling using Conformer–Rotamer Ensemble Sampling Tool (CREST) [79, 80, 81] in noncovalent interaction (NCI) mode. This exploration was carried out at the GFN2‐xTB [82, 83] level with implicit solvation described by the analytical linearized Poisson‐Boltzmann (ALPB) model for water [84]. Candidate structures within an energy window of 12 kcal/mol relative to the lowest GFN2‐xTB energy were retained, clustered by root mean square deviation (RMSD) to eliminate near‐duplicate structures, and ranked by GFN2‐xTB energy. The ten lowest‐energy representatives were then fully optimized at the DFT level (as described above), and BSSE‐corrected binding energies were computed for each DFT‐refined minimum *i*, denoted as *E*_bind,i_.

To obtain an ensemble‐averaged estimate within the sampled low‐energy set, we computed Boltzmann weights (ω_*i*_) at *T* = 300 K (Figure S9). We then reported the Boltzmann‐weighted mean binding energy as $\left\langle E_{\mathrm{bind}}\right\rangle =\sum_{i=1}^{10}\omega_{i}\;E_{\mathrm{bind},\;i}$ and the Boltzmann‐weighted standard deviation as $\sigma_{\mathrm{bind}}=\sqrt{\sum_{i=1}^{10}\omega_{i}{\left(E_{\mathrm{bind},\;i}-\left\langle E_{\mathrm{bind}}\right\rangle \right)}^{2}}$, which was used for the error bars in Figure 5H. We emphasize that the reported spread reflects variability across the finite set of DFT‐refined low‐energy minima obtained from the CREST‐based workflow within a continuum‐solvation description. These values, therefore, do not constitute a rigorous binding free energy; they do not include exhaustive configurational sampling at an explicit LC–aqueous interface, nor do they capture entropic contributions (and other free‐energy terms) beyond those implicit in the selected minima. Moreover, because the present DFT model is necessarily truncated to the charged headgroup region, it does not include the hydrophobic lipid tails and thus cannot represent tail‐mediated interfacial packing effects—such as aliphatic‐tail interdigitation, hydrophobic clustering, and associated interfacial restructuring—that may modulate complex stability and LC anchoring. Accordingly, the reported DFT binding energies should be interpreted primarily as comparative descriptors of relative binding strength (trends) within the chosen model and sampling protocol. A more rigorous quantification of interfacial binding free energies, including explicit ionic atmosphere/solvent fluctuations and tail‐driven packing/interdigitation at the LC interface, will require explicit‐interface ensemble averaging (e.g., molecular dynamics (MD)‐based approaches such as potential of mean force/umbrella sampling or alchemical free‐energy methods), which is beyond the present scope.

### 4.13 Local Energy Decomposition

To elucidate the physical origin of the interactions between the LPS/LTA headgroup and Glu^–^/Asp^–^, we performed single‐point DLPNO‐CCSD(T)/def2‐TZVP(‐f) calculations [85, 86] on the DFT‐optimized lowest‐energy binding geometries. Solvent effects were included using the CPCM model for water [87, 88, 89], and all DLPNO calculations employed the NormalPNO* settings. Occupied orbitals were localized using the Foster–Boys procedure [90]. The interaction energies were analyzed using the local energy decomposition (LED) scheme [63, 91, 92] as implemented in ORCA 6.1 [93].

Within the LED framework, the total interaction energy between two fragments Δ*E*_tot−int_ is decomposed as:

$$\Delta E_{\mathrm{tot}-\mathrm{int}}=\Delta E_{\mathrm{el}-\mathrm{prep}}+E_{\mathrm{elec}}+E_{\mathrm{exch}}+E_{\mathrm{disp}}+E_{\mathrm{non}-\mathrm{disp}}+E_{\mathrm{diel}}$$

Here, Δ*E*_el−prep_ is the (static) electronic preparation energy (i.e., the energy required to deform the isolated‐fragment electronic structures into those optimal for the interacting state), which is typically positive (repulsive). *E*_elec_ denotes the electrostatic interaction between the (prepared) fragment densities, while *E*_exch_ is the inter‐fragment exchange term, which is stabilizing by construction in the LED partitioning. Electron correlation further contributes through the London‐dispersion component (*E*_disp_) and the remaining non‐dispersion correlation term (*E*_non−disp_). Under the CPCM solvation model, an additional dielectric (solvation) term *E*_diel_ was reported [63].

To obtain functional‐group–resolved insight for the heterogeneously charged LPS/LTA headgroup, we partitioned the system into chemically meaningful fragments corresponding to major headgroup motifs (e.g., the Ara4N ammonium‐containing unit, sugar moieties, and phosphate‐containing groups) and the amino acid. Because some fragment definitions introduce covalent connections across fragment boundaries within the headgroup, we employed the covalent local energy decomposition (COVALED) treatment to properly handle covalently connected fragments in the LED analysis. Pairwise fragment–fragment interaction matrices were converted to fragment‐pairwise LED (fp‐LED) form [63] using the local energy decomposition analysis wizard (LEDAW) [94] and visualized as heat maps. This enables direct identification of the dominant stabilizing fragment–fragment channels and their component‐resolved contributions.

Finally, we note that LED decomposes the interaction energy, whereas binding energies additionally include geometric preparation (strain) contributions associated with bringing the fragments from their relaxed geometries to the bound geometry. Therefore, LED interaction energies are not expected to match the binding energies quantitatively, but they provide a mechanistically interpretable breakdown of the inter‐fragment interactions.

## Funding

This work was supported by the National Research Foundation of Korea (NRF) grant funded by the Korea government (MSIT) (RS‐2023‐00212739, RS‐2024‐00411809, 2022M3C1A3081312 and RS‐2023‐00302586).

## Conflicts of Interest

POSTECH has filed patent applications (10‐2024‐0151837, Korea; 19/343,198, USA) on the work described in this manuscript. The inventors listed on the patent application are Yena Choi, Hyunsoo Han, Sangmin Jeon, and Young‐Ki Kim.

## Supporting information




**Supporting File**: advs74816‐sup‐0001‐SuppMat.docx.

## Data Availability

The data that support the findings of this study are available in the supplementary material of this article.
