# Supplementary material for: Design of Optical Transducer for Recognition of Biomolecular Interactions between Bacterial Lipopolysaccharides and Amino Acids
Source: Adv Sci (Weinh). 2026 Mar 12;13(29):e23658. doi: 10.1002/advs.202523658 (PMC13205851; doi:10.1002/advs.202523658)
Supplement: Supplementary file 1 — Supporting File: advs74816‐sup‐0001‐SuppMat.docx. [file ADVS-13-e23658-s001.docx]

Supporting Information

Design of Optical Transducer for Recognition of Biomolecular Interactions between Bacterial Lipopolysaccharides and Amino Acids

Yena Choi, Sangmin Lee, Jin-Kang Choi, Hyunsoo Han, Yeongseon Choi, Jun-Hyung Im, Hyein Kim, Sangmin Jeon, Minjae Lee*, Chang Yun Son*, Young-Ki Kim*

Y. Choi, J.-K. Choi, H. Han, Y. Choi, J.-H. Im, H. Kim, Prof. S. Jeon, Prof. Y.-K. Kim

Department of Chemical Engineering, Pohang University of Science and Technology (POSTECH), Pohang 37673, Republic of Korea

E-mail: ykkim@postech.ac.kr

S. Lee, Prof. C. Y. Son

Department of Chemistry, Seoul National University, Seoul 08826, Republic of Korea

E-mail: changyunson@snu.ac.kr

Prof. M. Lee

Department of Chemistry, Kunsan National University, Gunsan 54150, Republic of Korea

E-mail: minjae@kunsan.ac.kr


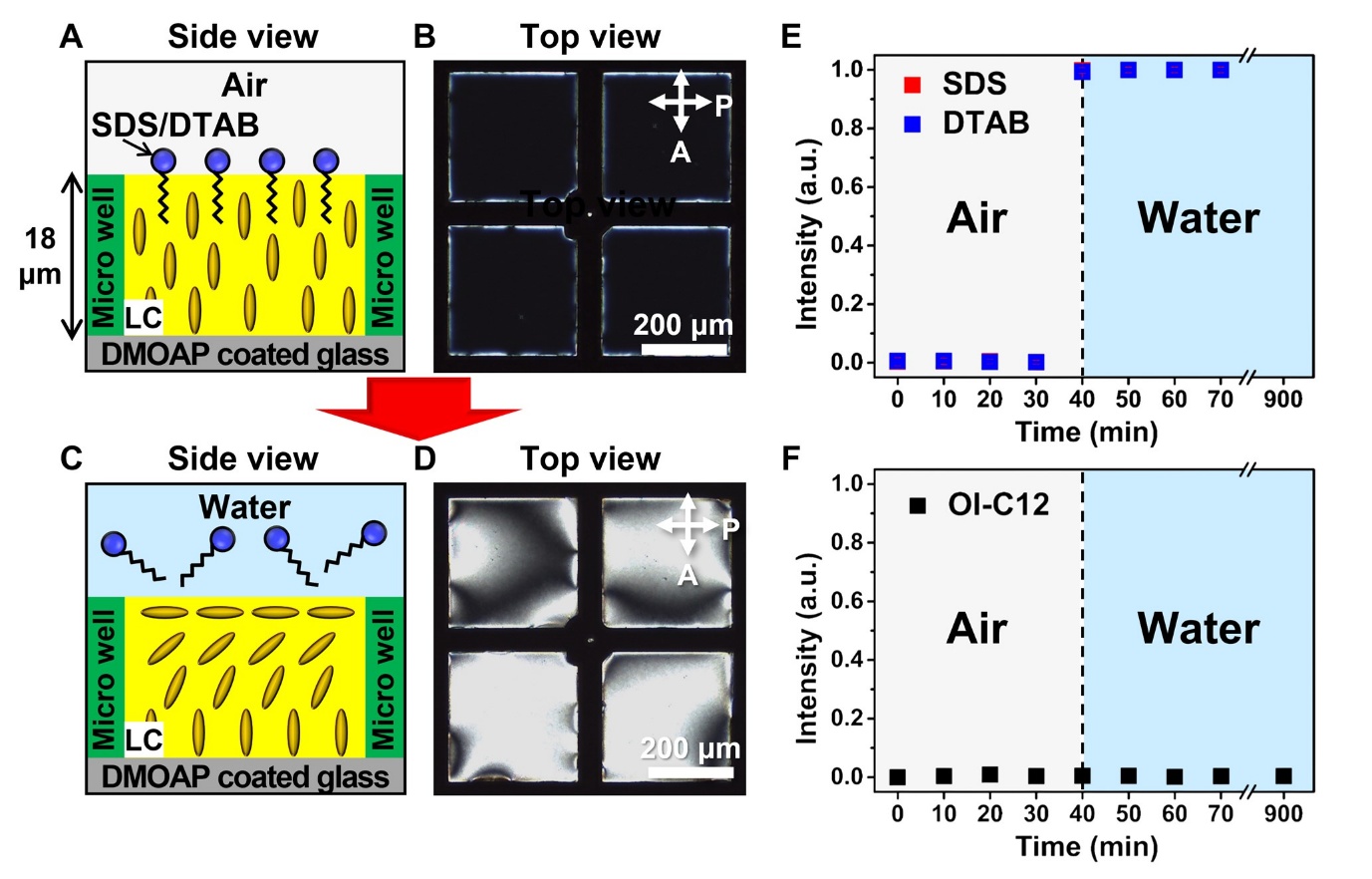


**Figure S1.** Desorption of SDS and DTAB from the LC-aqueous interface. (A-D) (A, C) Schematic illustrations of the LC film (*d* = 18 μm) with a reconstructed orientation of LC molecules, and (B, D) corresponding polarizing optical micrographs (A, B) before and (C, D) after water incubation. SDS or DTAB is initially doped into the LC phase at a concentration of 4.5 mM.^[S1]^ (E, F) Corresponding optical intensity profiles derived from (E) SDS- (red squares), DTAB- (blue squares), and (F) OI-C12 doped LC film (black squares). Before water incubation, all LC films assume the homeotropic configuration across the film thickness. When submerged in water, however, the SDS- or DTAB-doped LC films show an immediate dark-to-bright optical transition, while the OI-C12-doped LC film maintains the dark optical signal (i.e., homeotropic anchoring) for an extended period. These results imply the desorption of SDS or DTAB from the LC interface into the overlying water phase, whereas the OI-C12 remains stable at the LC-aqueous interface.


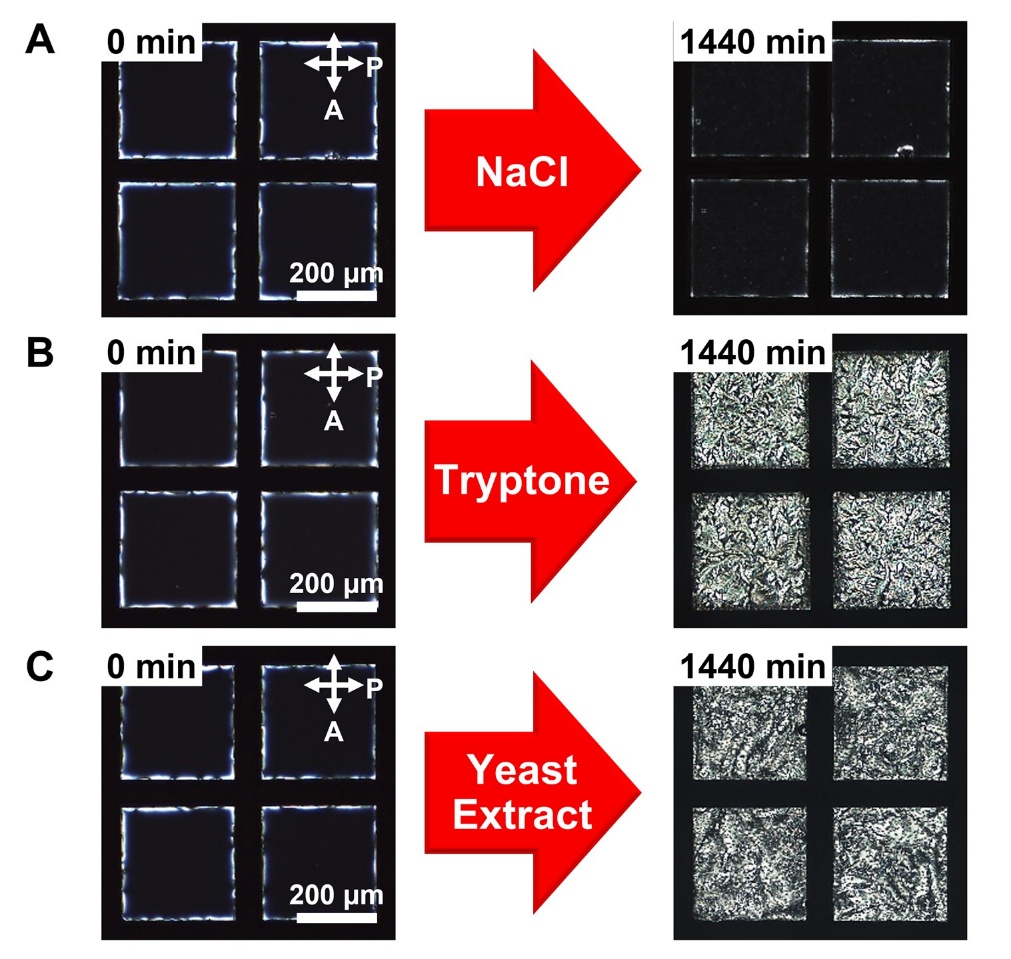


**Figure S2.** Optical transition of the homeotropic LC films in the aqueous solutions upon introduction of each component of the LB broth. (A-C) Optical micrographs of the homeotropic thick LC film (*d* = 18 μm, with **OI-C12**) in the aqueous solutions before (left) and after (right) the introduction of (A) sodium chloride, (B) tryptone, and (C) yeast extract. LB broth consists of 10 g/L of sodium chloride, 10 g/L of tryptone, and 5 g/L of yeast extract.^[S2]^ The injected concentration of each component is equivalent with that in the W-LB solution (*C*_LB_ = 16.7% (v/v)). We observe the optical transition of LC films only upon the introduction of tryptone and yeast extract, and find their transition behavior (e.g., transition rate, intensity) to be noticeably similar. Therefore, as key determinants to trigger the anchoring/optical transition of LCs, we focus on amino acids which are the common components present both in tryptone and yeast extract.


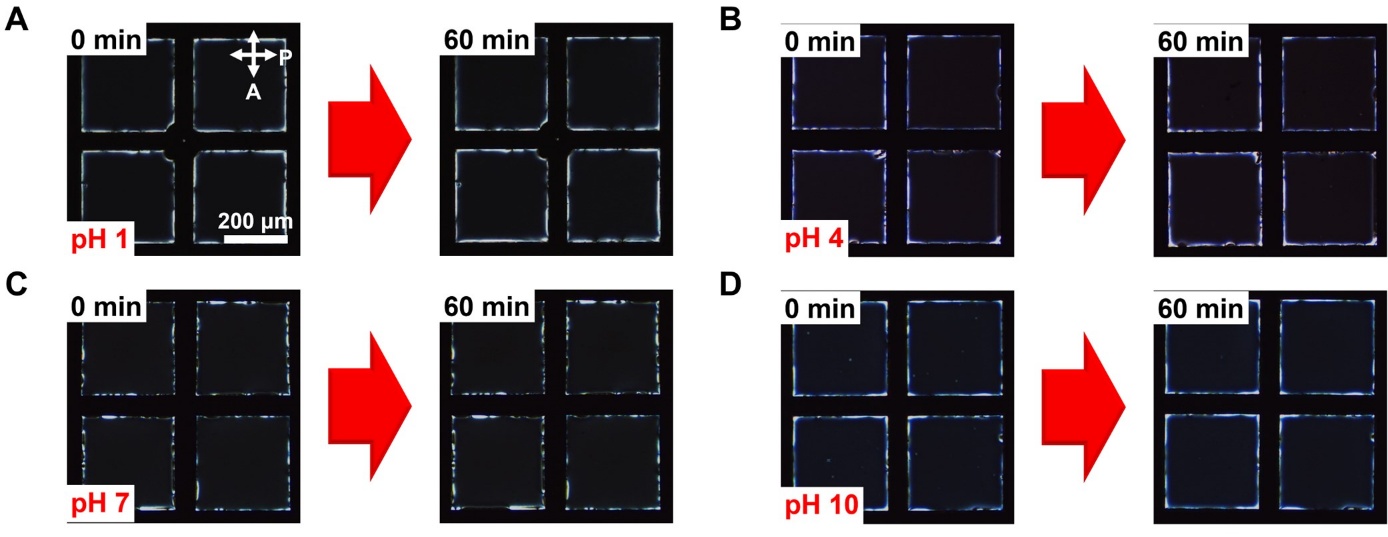


**Figure S3.** No optical response of the LC films in different aqueous pH buffer solutions. (A-D) Optical micrographs of the thick LC films (*d* = 18 μm, with **OI-C12**) in the aqueous pH butter solutions at (A) pH 1, (B) pH 4, (C) pH 7, and (D) pH 10. The micrographs are taken at 0 (left) and 60 min (right) after the incubation


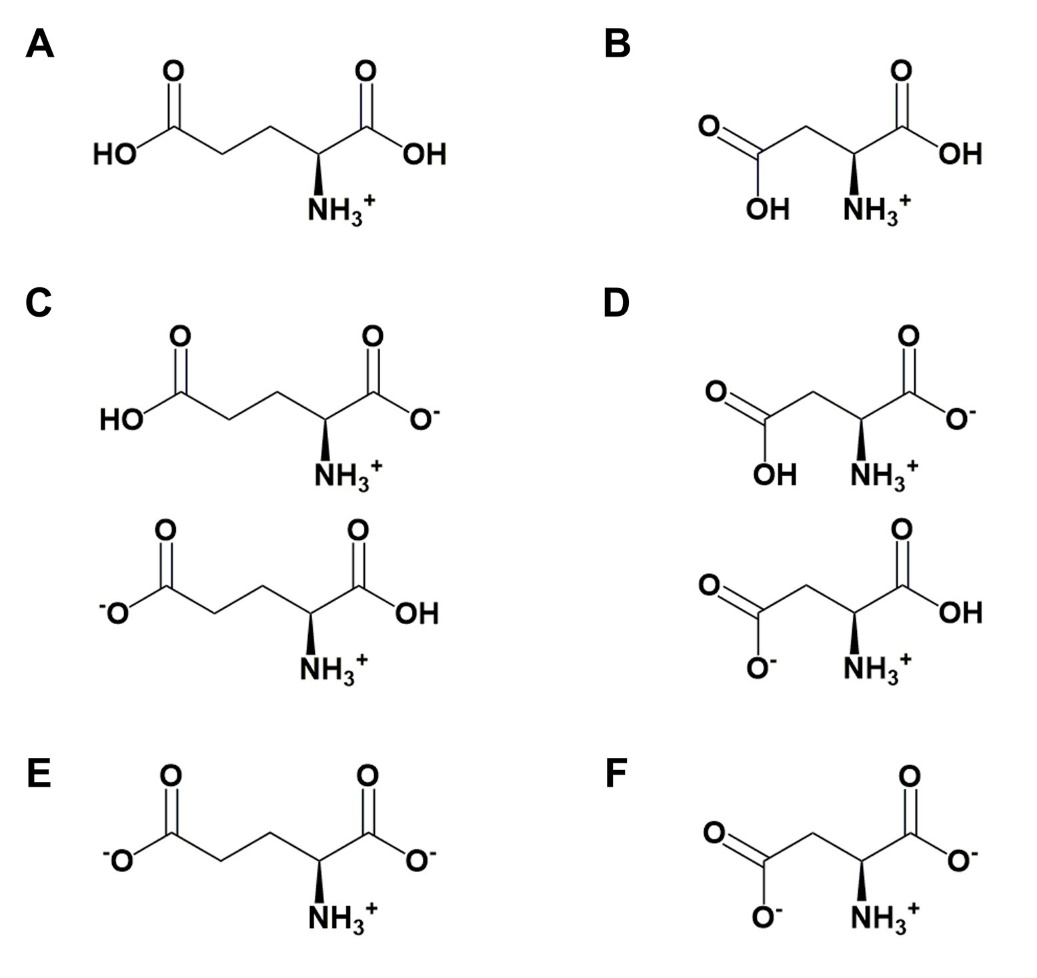


**Figure S4.** Charged states of Glu and Asp in aqueous solutions with different pH conditions. (A, B) Positively charged states of (A) Glu^+^ and (B) Asp^+^, which are dominantly present at pH 1.5. (C, D) Neutrally charged states of (C) Glu^0^ and (D) Asp^0^, which are dominantly present at pH 3.6. (E, F) Negatively charged states of (E) Glu^–^ and (F) Asp^–^, which are dominantly present at pH 6.2.


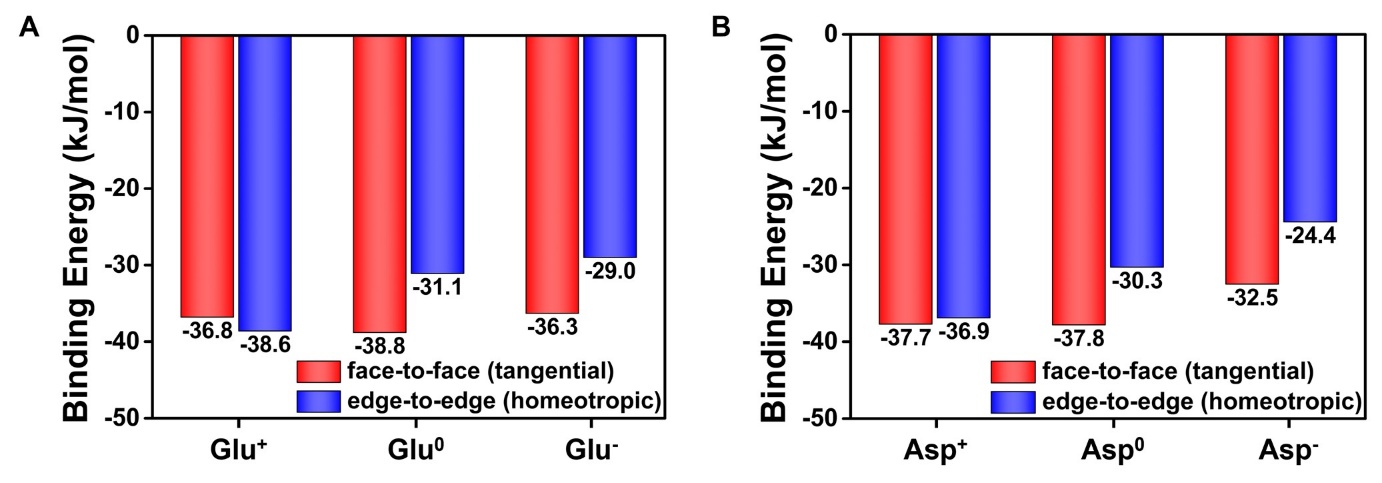


**Figure S5.** DFT-calculated binding energies for the different charge states of Glu and Asp with the nematic LC 5CB in the aqueous phase. (A, B) Binding energies of cationic, neutral, and anionic forms of (A) Glu and (B) Asp interacting with 5CB in the face-to-face (red bars) and edge-to-edge (blue bars) configurations.


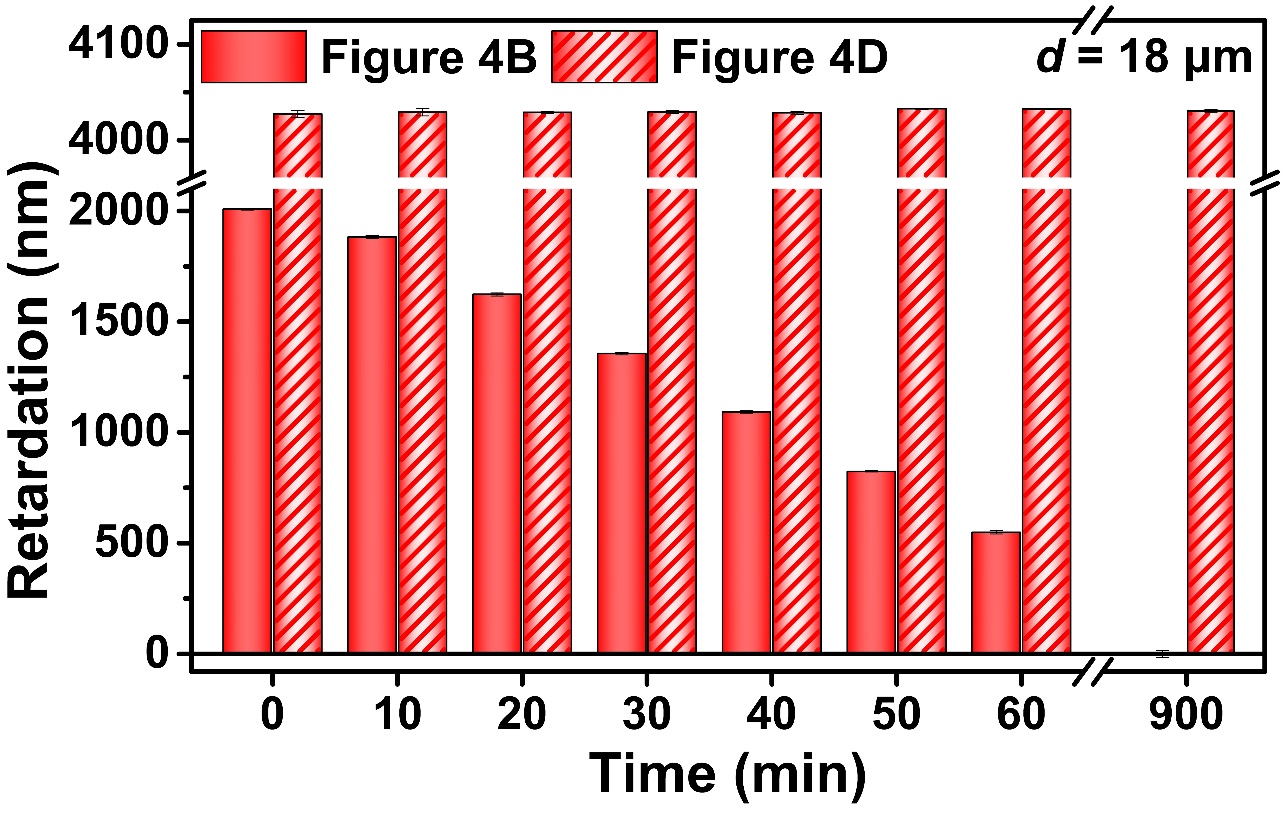


**Figure S6.** Time-dependent *Γ*_m_ of homeotropic (with **OI-C12**, solid-red bars**)** and tangential (no **OI-C12**, dashed-red bars) thick LC films in the W-[Glu+Asp] solution. The measurements are initiated after the LC films reach their maximum *Γ*_m_ (i.e., the states in Figure 4B and Figure 4D). Under prolonged incubation in the W-[Glu+Asp] solution, the homeotropic LC film with hybrid configuration (solid-red bar at 0 min) exhibits a gradual reduction in *Γ*_m_ and eventually restores the initial homeotropic configuration (Fig. 4A, *Γ*_m_ = 0 nm at 900 min). This observation is consistent with the transient optical transition (solid-pink line in Figure 2F), further supporting the interfacial adsorption and subsequent desorption of Glu and Asp. In contrast, the tangential LC film (no **OI-C12**, dashed-red bars) maintains a stable *Γ*_m_ of 4,030 $\pm$ 3 nm over the time period of 900 min. The stable tangential anchoring of LCs is governed by the LC-aqueous without **OI-C12**, remaining independent of the interfacial adsorption and desorption dynamics of Glu and Asp.


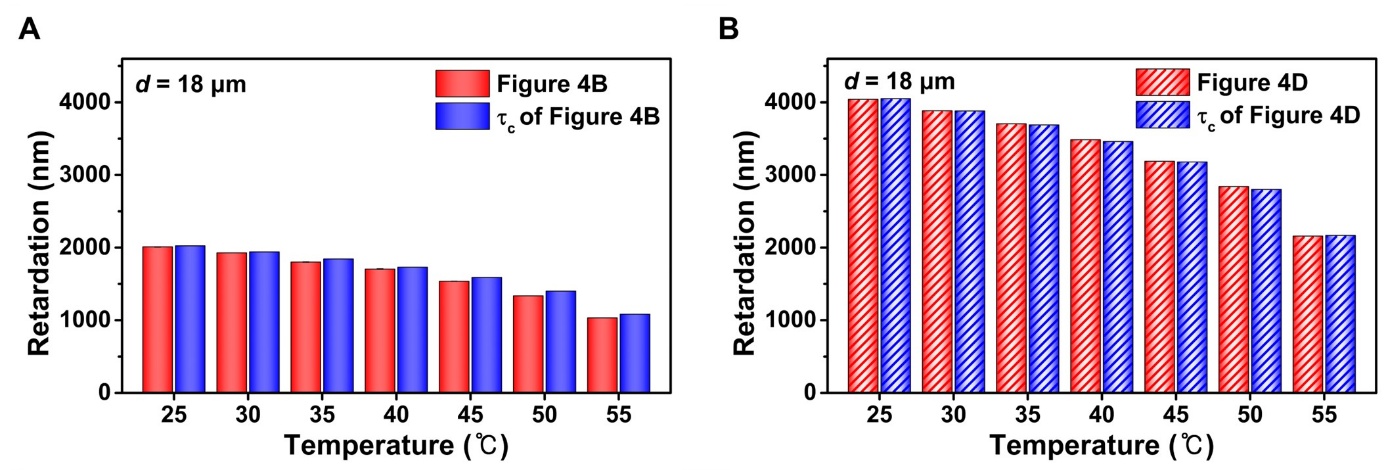


**Figure S7.** *T*-dependent optical retardation of LC films. (A) Retardation values measured (solid-red bars) and calculated (solid-blue bars) from the homeotropic thick LC films (with **OI-C12**) with the hybrid configuration in W-[Glu+Asp] solution (Figure 4B). (B) Retardation values measured (dashed-red bars) and calculated (dashed-blue bars) from the tangential thick LC films (no **OI-C12**) in W-[Glu+Asp] solution (Figure 4D). In the calculation of retardation (*Γ*_c_), we use the temperature-dependent birefringence values of E7.


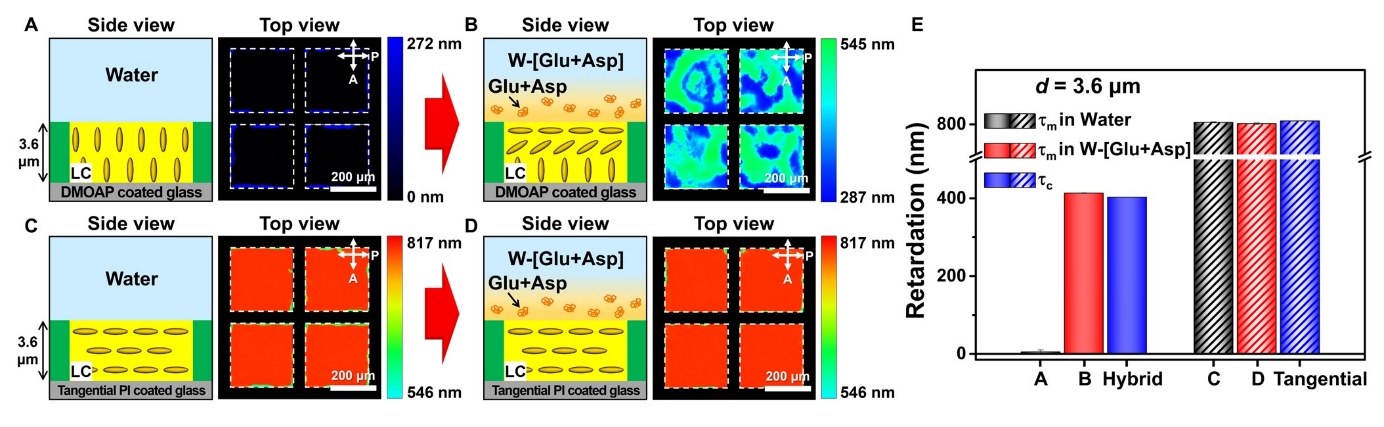


**Figure S8**. Amino acids-induced easy axis reorientation of LCs revealed by retardation measurements from the thin LC films. (A-D) Schematic illustrations (side view) of (A, B) the homeotropic and (C, D) tangential thin LC films (*d* = 3.6 μm, no OI-C12), and corresponding retardation color maps (top view) in (A, C) the pure water and (B, D) W-[Glu+Asp] solutions. The retardation color maps in (B) and (D) are taken when the retardation reaches the maximum value. (E) Retardation values measured (*Γ*_m_, black and red bars) from (A-D) and calculated (*Γ*_c_, blue bars) from the thin LC films with the hybrid and tangential configurations corresponding to the reconstructed LC ordering in (B) and (D), respectively.


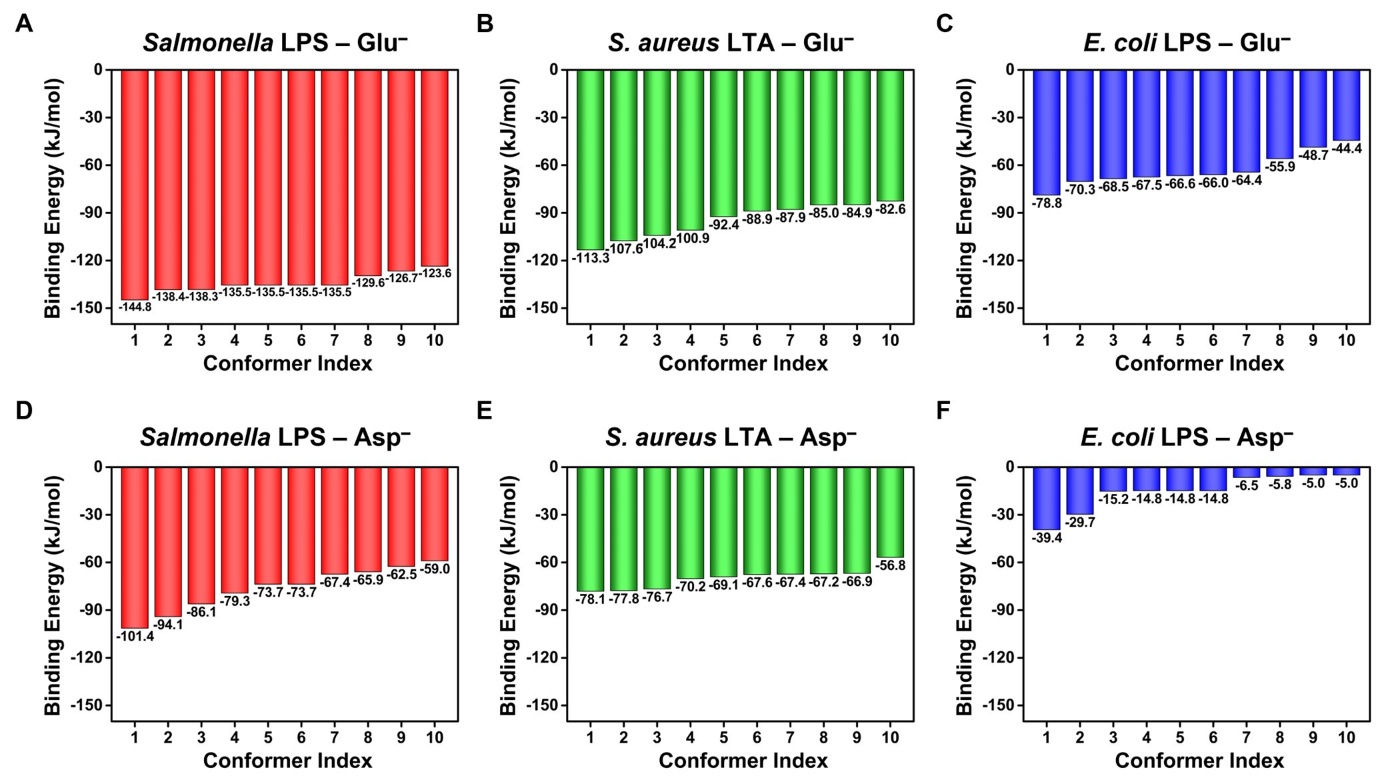


**Figure S9.** DFT-calculated binding energies (kJ/mol) for the ten lowest-energy DFT-refined minima of Glu^–^/Asp^–^ with S-LPS, SA-LTA, and EC-LPS. The top row shows Glu^–^ complexes and the bottom row shows Asp^–^ complexes with (A, D) S-LPS, (B, E) SA-LTA, and (C, F) EC-LPS. The y-axis range is identical to that in Figure 5H.


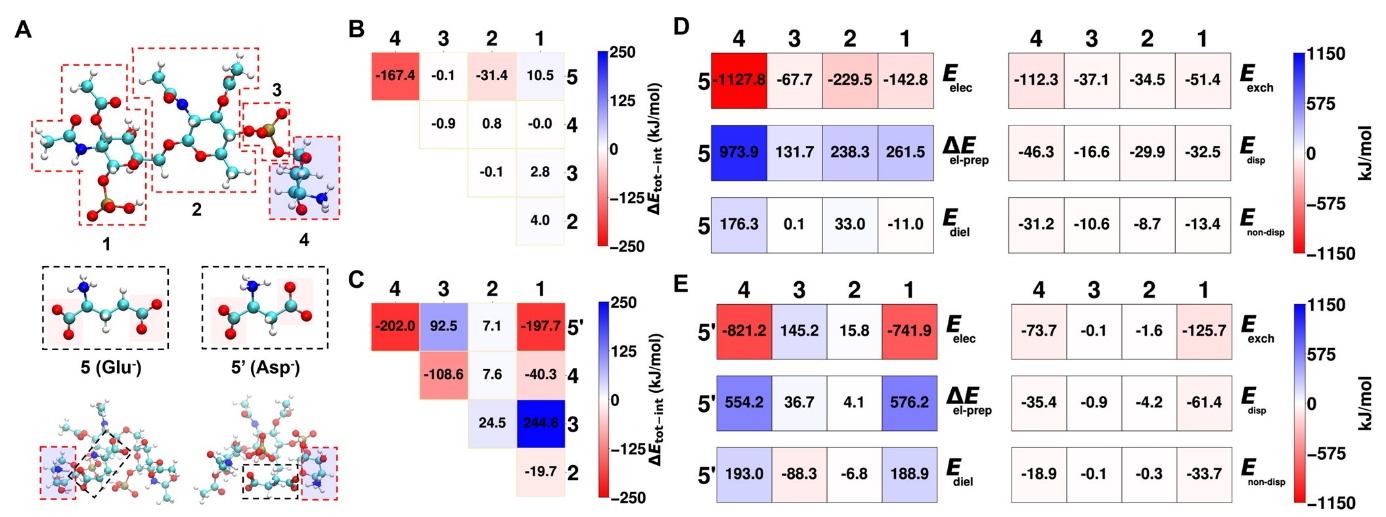


**Figure S10.** Fragment-pairwise local energy decomposition (fp-LED) analysis for S-LPS headgroup interaction with Glu^–^/Asp^–^. (A) Fragment definitions for the S-LPS headgroup (fragments 1-4) and the anionic amino acid (fragment 5 for Glu^–^, fragment 5’ for Asp^–^). (B, C) fp-LED interaction maps for the S-LPS complexes with (B) Glu⁻ and (C) Asp⁻. (D, E) Corresponding component-resolved fp-LED interaction maps partitioning the total inter-fragment interaction energy into electronic preparation (Δ*E*_el-prep_), electrostatics (*E*_elec_), dielectric (*E*_diel_), exchange (*E*_exch_), dispersion (*E*_disp_), and non-dispersion (*E*_non-disp_) contributions for the S-LPS complexes with (D) Glu⁻ and (E) Asp⁻. Negative values (red) indicate stabilizing/attractive contributions, whereas positive values (blue) indicate destabilizing/repulsive contributions. The decomposition identifies electrostatics as the dominant stabilizing term, most prominently between the Glu⁻/Asp⁻ carboxylate and the Ara4N ammonium-containing fragment. Furthermore, additional stabilizing contacts involve phosphate-containing fragments in the S-LPS complex with Asp⁻.


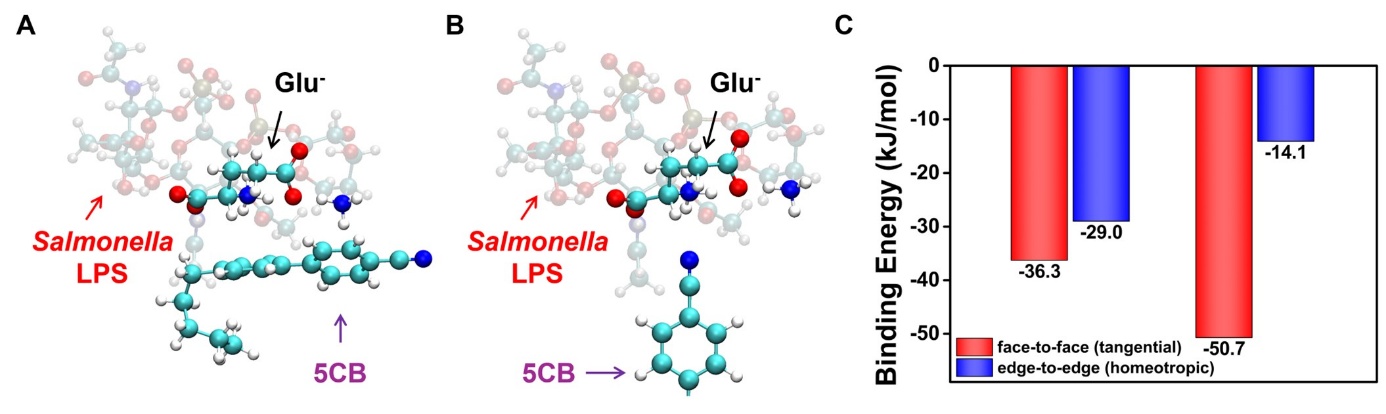


**Figure S11.** Complex formation of S-LPS–Glu⁻ with LC 5CB in the aqueous phase. (A, B) DFT-optimized geometries of the S-LPS–Glu⁻ complex interacting with 5CB in the face-to-face (tangential; A) and edge-to-edge (homeotropic; B) configurations. In the face-to-face configuration, the primary Glu⁻–5CB contact is largely retained, while the additional ammonium moiety in S-LPS enables closer association with the aromatic core of 5CB via cation–π interaction. (C) DFT-calculated binding energies of Glu⁻ and S-LPS–Glu⁻ interacting with 5CB for the face-to-face (red bars) and edge-to-edge (blue bars) configurations, showing that S-LPS complexation strengthens face-to-face binding but weakens edge-to-edge binding, thereby amplifying the orientation selectivity.


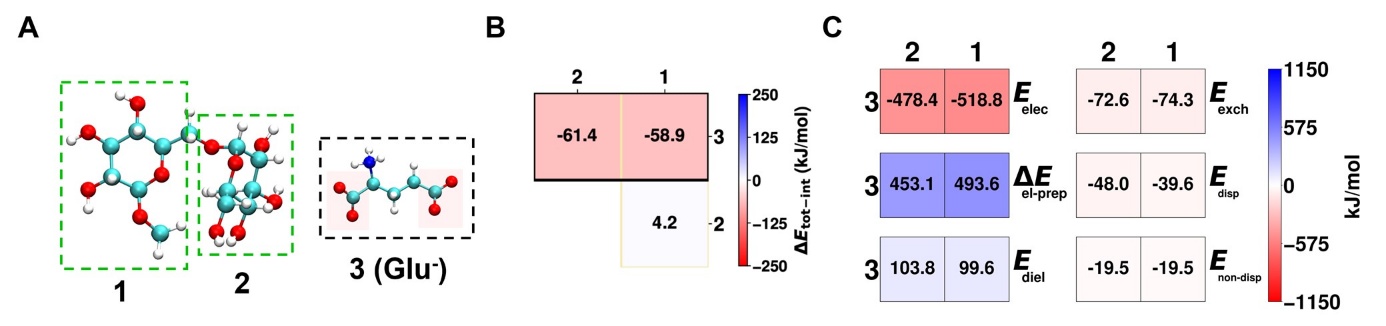


**Figure S12.** Fragment-pairwise local energy decomposition (fp-LED) analysis of the SA-LTA headgroup interaction with Glu⁻. (A) Fragment definitions used for the SA-LTA headgroup (fragments 1–2) and Glu⁻ (fragment 3). (B) fp-LED interaction map for the SA-LTA complex with Glu⁻. (C) Corresponding component-resolved fp-LED interaction maps partitioning the total inter-fragment interaction energy into electronic preparation (Δ*E*_el-prep_), electrostatics (*E*_elec_), dielectric (*E*_diel_), exchange (*E*_exch_), dispersion (*E*_disp_), and non-dispersion (*E*_non-disp_) contributions. Negative values (red) indicate stabilizing/attractive contributions, whereas positive values (blue) indicate destabilizing/repulsive contributions. The decomposition identifies electrostatics as the dominant stabilizing term. The color-bar range is identical to that used in Figure S10.


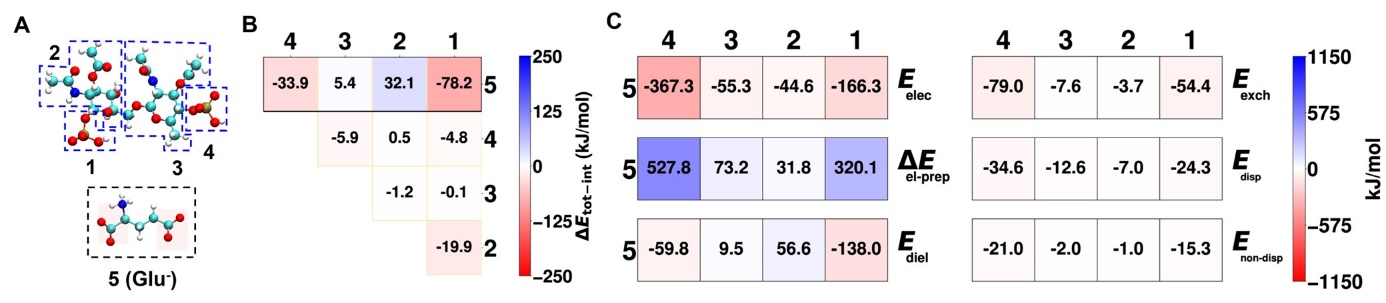


**Figure S13.** Fragment-pairwise local energy decomposition (fp-LED) analysis of the EC-LPS headgroup interaction with Glu⁻. (A) Fragment definitions for the EC-LPS headgroup (fragments 1–4) and Glu⁻ (fragment 5). (B) fp-LED interaction map for the EC-LPS complex with Glu⁻. (C) Corresponding component-resolved fp-LED interaction maps partitioning the total inter-fragment interaction energy into electronic preparation (Δ*E*_el-prep_), electrostatics (*E*_elec_), dielectric (*E*_diel_), exchange (*E*_exch_), dispersion (*E*_disp_), and non-dispersion (*E*_non-disp_) contributions. Negative values (red) indicate stabilizing/attractive contributions, whereas positive values (blue) indicate destabilizing/repulsive contributions. The decomposition identifies electrostatics as the dominant stabilizing term. The color-bar range is identical to that used in Figure S10.


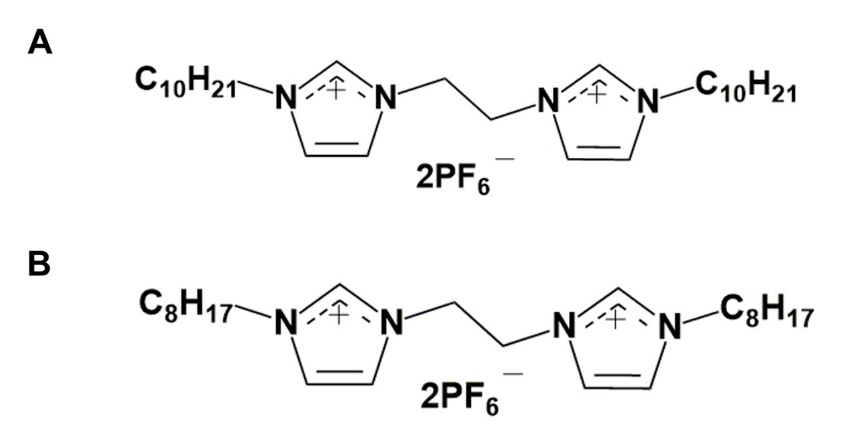


**Figure S14.** Molecular structures of OI-C10 and OI-C8. (A, B) Molecular structures of (A) **OI-C10** and (B) **OI-C8**.

**
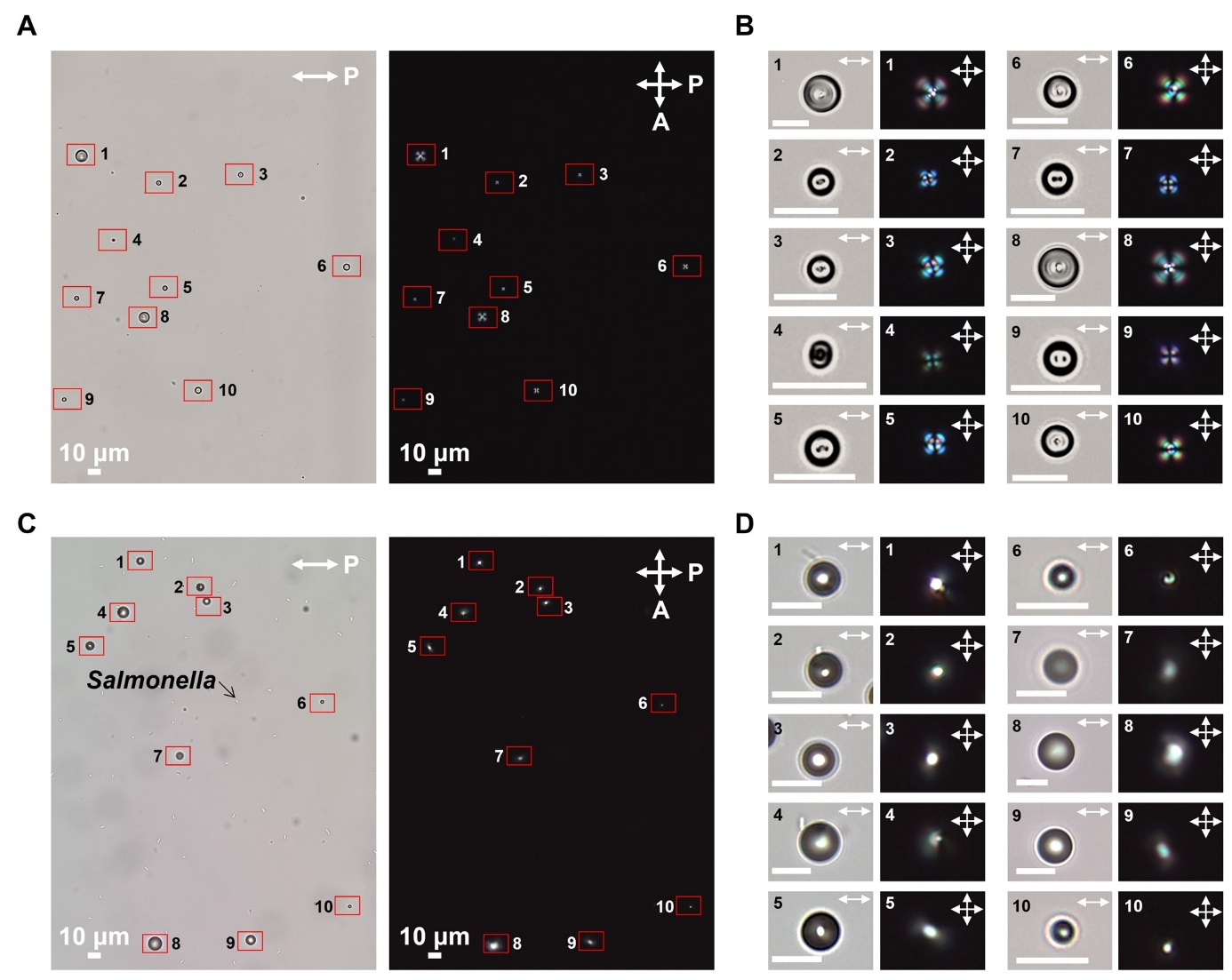
**

**Figure S15.** Optical response of LC droplets. (A-D) POM images for LC droplets treated with **OI-C12** in (A, B) pure water and (C, D) the W-LB-S (*C*_LB_ = 16.7% (v/v) and *C_S_* ~ 10^2^ cfu/ml) solutions. (B) and (D) show magnified views of the LC droplets, as indicated by the red boxes in (A) and (C), respectively.

**Supplemental References**

S1. H. Tan, X. Li, S. Liao, et al., "Highly-sensitive liquid crystal biosensor based on DNA dendrimers-mediated optical reorientation," Biosensors and Bioelectronics 62, (2014): 84-89. https://doi.org/10.1016/j.bios.2014.06.029

S2. M. P. MacWilliams and M. K. Liao, "Luria broth (LB) and Luria agar (LA) media and their uses protocol," *ASM*, (2006): 1-4.
